# Clinical Subtype Trajectories in Sepsis Patients Admitted to the ICU: A Secondary Analysis of an Observational Study

**DOI:** 10.1097/CCE.0000000000001176

**Published:** 2024-11-14

**Authors:** Marleen A. Slim, Rombout B. E. van Amstel, Marcella C.A. Müller, Olaf L. Cremer, Alexander P. J. Vlaar, Tom van der Poll, W. Joost Wiersinga, Christopher W. Seymour, Lonneke A. van Vught

**Affiliations:** 1 Department of Intensive Care, Amsterdam University Medical Center, University of Amsterdam, Amsterdam, The Netherlands.; 2 Center for Experimental and Molecular Medicine, Amsterdam University Medical Center, University of Amsterdam, Amsterdam Institute for Infection and Immunity, Amsterdam, The Netherlands.; 3 Department of Intensive Care Medicine, University Medical Center Utrecht, Utrecht, The Netherlands.; 4 Department of Medicine, Division of Infectious Diseases, Amsterdam University Medical Center, University of Amsterdam, Amsterdam, The Netherlands.; 5 Department of Critical Care Medicine, School of Medicine, University of Pittsburgh, Pittsburgh, PA.; 6 Department of Emergency Medicine, School of Medicine, University of Pittsburgh, Pittsburgh, PA.; 7 Clinical Research, Investigation, and Systems Modeling of Acute Illness Center, School of Medicine, University of Pittsburgh, Pittsburgh, PA.

**Keywords:** biomarkers, phenotypes, sepsis, subtypes, trajectories

## Abstract

**OBJECTIVES:**

Sepsis is an evolving process and proposed subtypes may change over time. We hypothesized that previously established sepsis subtypes are dynamic, prognostic of outcome, and trajectories are associated with host response alterations.

**DESIGN:**

A secondary analysis of two observational critically ill sepsis cohorts: the Molecular diAgnosis and Risk stratification of Sepsis (MARS) and the Medical Information Mart for Intensive Care-IV (MIMIC-IV).

**SETTING:**

ICUs in the Netherlands and United States between 2011–2014 and 2008–2019, respectively.

**PARTICIPANTS:**

Patient admission fulfilling the Sepsis-3 criteria upon ICU admission adjudicated to one of four previously identified subtypes, comprising 2,416 admissions in MARS and 10,745 in MIMIC-IV.

**MAIN OUTCOMES AND MEASURES:**

Subtype stability and the changes per subtype on days 2, 4 and 7 of ICU admission were assessed. Next, the associated between change in clinical subtype and outcome and host response alterations.

**RESULTS:**

In MARS, upon ICU admission, 6% (*n* = 150) of the patient admissions were α-type, 3% (*n* = 70) β-type, 55% (*n* = 1317) γ-type, and 36% (*n* = 879) δ-type; in MIMIC-IV, this was α = 22% (*n* = 2398), β = 22% (*n* = 2365), γ = 31% (*n* = 3296), and δ = 25% (2686). Overall, prevalence of subtypes was stable over days 2, 4, and 7. However, 28–56% (MARS/MIMIC-IV) changed from α on ICU admission to any of the other subtypes on day 2, 33–71% from β, 57–32% from γ, and 50–48% from δ. On day 4, overall subtype persistence was 33–36%. γ or δ admissions remaining in, or transitioning to, subtype γ on days 2, 4, and 7 exhibited lower mortality rates compared with those remaining in, or transitioning to, subtype δ. Longitudinal host response biomarkers reflecting inflammation, coagulation, and endothelial dysfunction were most altered in the δ-δ group, followed by the γ-δ group, independent of the day or biomarker domain.

**CONCLUSIONS AND RELEVANCE:**

In two large cohorts, subtype change to δ was associated with worse clinical outcome and more aberrant biomarkers reflecting inflammation, coagulation, and endothelial dysfunction. These findings underscore the importance of monitoring sepsis subtypes and their linked host responses for improved prognostication and personalized treatment strategies.

**KEY POINTS**

**Question:** Are previously established sepsis subtypes dynamic over time, prognostic of outcome and associated with host response alterations?

**Findings:** This cohort study demonstrates that previously established sepsis subtypes change over time and are associated with outcome and host response alterations. Sepsis subtype prevalence remained relatively stable throughout the first week of ICU admission, yet individual patients quite often transitioned between subtypes over time. Patients remaining or transitioning to subtype γ were associated with lower mortality rates compared with patients remaining or switching to δ.

**Meaning:** These findings underscore the importance of monitoring sepsis subtypes for improved prognostication and personalized treatment strategies.

Sepsis is a heterogeneous syndrome characterized by a dysregulated immunological response to an infection resulting in organ dysfunction and often mortality (1). Despite extensive research, treatment for sepsis primarily includes source control, administration of antibiotics, and organ support (2). Sepsis exhibits a highly heterogeneous clinical presentation, treatment response, and prognosis due to a wide-range of causing pathogens, infections sites, and host factors such as comorbidities, age, and genetic composition (3). Many sepsis subtypes or sepsis immune states are proposed, with plausible differential response to treatments (4–7). Utilizing clinical data, four distinct sepsis subtypes (designated as α, β, γ, and δ) were identified upon presentation to the emergency department through an unbiased clustering technique (8). These subtypes exhibited differences in clinical characteristics and outcomes. Importantly, when varying the subtype frequency included in randomized controlled trials in sepsis, the outcome of the trials changed considerably, indicating that these subtypes may be relevant for treatment responses (8). Given that sepsis is a dynamic process due to disease progression and treatment response, time could be of important influence to subtype membership and patients might change subtypes over time. However, whether a change in subtype over time could be prognostic of outcome and predictive of treatment response is still to be determined.

Several approaches to model sepsis over time have been studied and associated with clinical outcome, for example, using group-based trajectory modeling of repeated temperature measurements (9), vital sign (10), and organ dysfunction trajectories (11). Nevertheless, merging these with machine-learned subtypes and incorporating them into clinical trials and/or use is difficult because of the lack of: 1) available data on suitable measurements, 2) knowledge about the influence of treatment interventions on natural trajectories, and 3) availability of longitudinal biological measurement in sepsis patients combined with their corresponding subtypes over time (7, 12). A greater understanding of how subtypes and their linked biological pathways change, could lead to the identification of a treatable trait, defined as a biological abnormality of interest that has a predictable response to therapy, which then might lead to improvements in sepsis management (7).

To address this knowledge gap, we sought to determine if previously established sepsis subtypes (8) are dynamic over time, prognostic of outcome and associated with host response alterations.

## MATERIALS AND METHODS

### Patients

This study was conducted in two cohorts: 1) in the Molecular diAgnosis and Risk stratification of Sepsis (MARS) cohort, a prospective observational cohort study in the mixed ICUs of two tertiary teaching hospitals (Amsterdam Universal Medical Center in Amsterdam and University Medical Center Utrecht in Utrecht) and 2) in the Medical Information Mart for Intensive Care-IV (MIMIC)-IV database (13, 14) containing publicly available retrospective observational data of patients admitted to the ICUs of the Beth Israel Deaconess Medical Center, Boston Massachusetts between 2008 and 2019 (15). In the current analyses, the MARS cohort was used as discovery cohort and the MIMIC-IV cohort as validation cohort. The MARS study included all patients above 18 years old who were admitted to the ICU having an expected length of stay longer than 24 hours between January 2011 and January 2014. Patients were included via an opt-out method approved by the medical ethical committees of the participating hospitals. In this secondary analysis, only patients fulfilling the Sepsis-3 criteria (1) were included (**Supplementary Methods**, http://links.lww.com/CCX/B432). ICU readmissions within the same hospital admission or within 30 days after ICU admission were excluded. In MIMIC-IV, sepsis patients were identified using Sepsis-3 criteria, as the suspicion of an infection in combination with a Sequential Organ Failure Assessment (SOFA) score of two or more at admission. The code of data extraction is available on GitHub (https://github.com/MIT-LCP/mimic-iv/concepts/sepsis/sepsis3.sql). Patients readmitted to the ICU within the same hospital admission were excluded. In both cohorts, patient admissions were excluded if greater than or equal to 50% of the variables needed for the adjudication of the subtypes was missing at the days the subtypes were allocated.

### Adjudication to Clinical Subtypes

Sepsis subtype variables were queried for missingness, converted to align with the variable units in Sepsis Endotyping in Emergency Care (SENECA) cohort (the cohort used for the establishment of the sepsis subtypes (8)), and the highest or lowest value (depending on the variable) was selected (Supplementary Methods, http://links.lww.com/CCX/B432). Data were analyzed for parametric distribution by Shapiro-Wilk tests and histogram plots and normalized as appropriate. Missing data were checked and only variables with less than 80% missing at admission were used for subtype adjudication (**Supplementary Figs. 1–4**, http://links.lww.com/CCX/B432). Missing was assumed to be “missing at random” and imputed using a flexible multivariable imputation procedure of multiple chained regression equations with predictive mean matching (16). The imputation cohort contained all available daily variables from ICU admission until day 7. A total of five datasets were generated, of which one dataset was randomly selected to carry forward. The clinical subtypes were adjudicated using the Euclidean distance from each patient to the centroid of each subtype from the SENECA cohort (17). Subtypes were mapped to data on ICU admission, day 2, day 4, and day 7.

### Host Response Biomarkers

In the MARS cohort, a subset of consecutive patient admissions with sepsis between January 2011 and July 2013 were selected for host response biomarker analysis. EDTA anti-coagulated plasma was obtained within 16 hours after ICU admission (day 0–1) and on day 2, day 4, and days 6–8. Biomarker panels measured consisted of angiopoietin-1, angiopoietin-2, d-dimer, interleukin (IL)-6, IL-8, IL-10, fractalkine, matrix metalloproteinase-8 (MMP-8), protein C, soluble E-selectin, and soluble intercellular adhesion molecule-1 (Supplementary Methods, http://links.lww.com/CCX/B432).

### Outcomes

For both cohorts, subtype stability was analyzed as the percentage of overall change from admission subtype to any of the other subtypes on days 2, 4, and 7. Next, the changes per subtype on days 2, 4, and 7 of ICU admission were assessed and compared. The secondary outcomes were to investigate if a change in clinical subtype is associated with: 1) outcome (30-d mortality, 1-yr mortality, ICU mortality, hospital mortality, and length of ICU stay) and 2) host response alterations.

### Statistical Analysis

Patient characteristics and outcomes were compared using a *t* test or analysis of variance for parametric data, Mann-Whitney *U* or Kruskal-Wallis test for nonparametric data, and a chi-square test for categorical data, stratified per subtype. Subtype distribution and transition of subtypes on ICU admission, days 2, day 4, and day 7 were depicted using alluvial plots. Furthermore, it was assessed which variables determined if a patient switches subtypes or stays within the same subtype; for more details, please see Supplementary Methods (http://links.lww.com/CCX/B432). Association of subtype dynamics with outcome and host response alterations was investigated in the two most frequent subtypes in the MARS, δ and γ (17), among patients who had remained in the ICU for greater than or equal to 2 days. Patients were grouped into subtype at admission and day 2 as follows: γ-γ, γ-δ, δ-γ, and δ-δ. The association between ICU admission and day 2 (vs. day 4 and/or 7) was chosen since information on subtype change on day 2 was deemed of more clinical relevance and since the median length of stay on the ICU was 3 days. Survival was visualized using Kaplan-Meier curves and analyzed using Cox regression if proportional hazards were met; otherwise, a logistic regression was employed. An unadjusted analysis was performed since variables suitable for adjustment were used to allocate the subtypes. In the first sensitivity analysis, 30-day mortality was also compared in α-α to α-γ and α-δ and in β-β to β-γ and β-δ (admission-day 2) to investigate whether transitioning from α or β to γ or δ would lead to similar results as transitioning from γ or δ to δ or γ. In the second sensitivity analysis, 30-day mortality was also compared in patients grouped into subtype at admission and day 4 and at admission and day 7 in patients with δ-γ vs. δ-δ and γ-δ vs. γ-γ. The longitudinal change of the host response biomarkers was estimated using a linear mixed model. Subtype on ICU admission-day 2, sample day, and their interaction were included as fixed effects. Random intercepts were assigned to each subject. A two sided *p* value of less than 0.05 was considered of statistical significance.

## RESULTS

### Population

**Figure 1.**
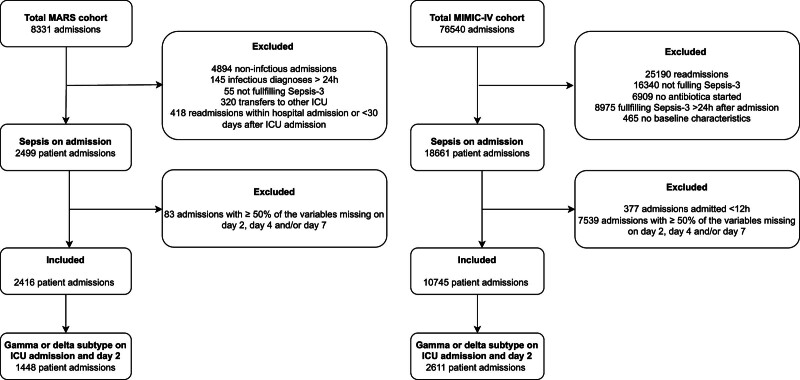
Flowchart of patient selection. Flowchart of patient selection in the Molecular diAgnosis and Risk stratification of Sepsis (MARS) and Medical Information Mart for Intensive Care-IV (MIMIC-IV) cohort.

Of the 8331 admissions included in the MARS cohort, 2416 patient admissions (29%) fulfilled all inclusion criteria and were used in this analysis (**Fig. 1**). In MIMIC-IV, of 76,540 admissions, 10,745 patient admissions (14%) were eligible. Patient admissions from both cohorts had a similar age (median, 63 yr; interquartile range [IQR], 51–72 yr for MARS and 67 yr [IQR, 55–79 yr] for MIMIC-IV) and gender (male 61% vs. 58%; **Supplementary Table 1**, http://links.lww.com/CCX/B432). On ICU admission, the median SOFA score was higher in MARS (7 [IQR, 4–9]) compared with MIMIC-IV (3 [IQR, 2–5]). The 30-day mortality was 26% in MARS and 20% in MIMIC-IV.

### Subtype Trajectories

**Figure 2.**
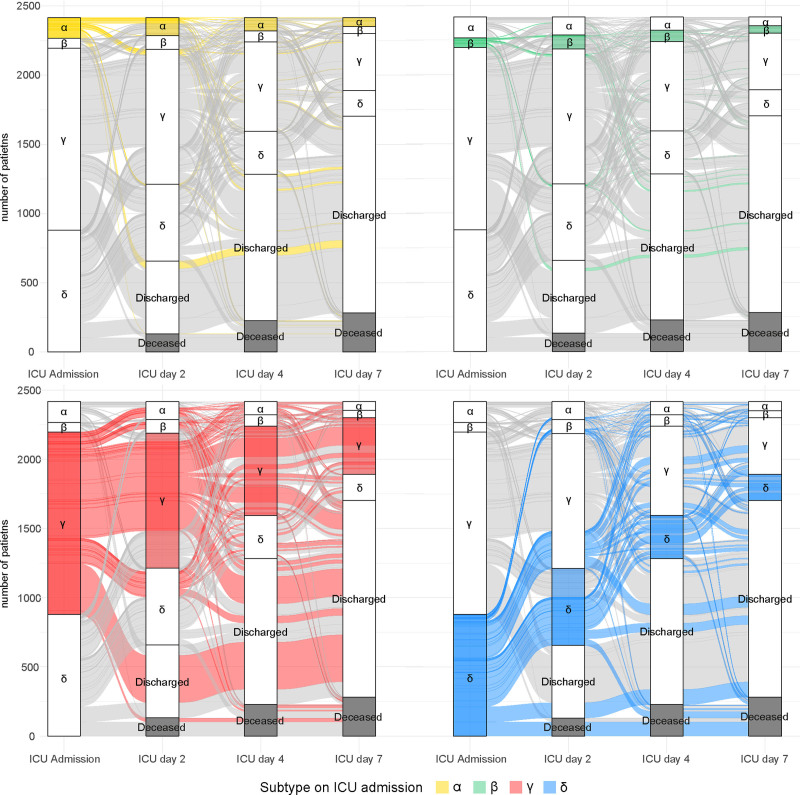
Distribution of subtypes in the Molecular diAgnosis and Risk stratification of Sepsis (MARS) cohort. Distribution of subtypes displayed on ICU admission, days 2, 4, and 7 in the MARS cohort. Subtype distribution in sepsis patients admitted to the ICU displayed per subtype at admission in the MARS cohort. At admission, 150 patients (6.2%) has the α subtype (*top left*, *yellow*), 70 patients (2.9%) β (*top right*, *green*), 1317 (54.5%) γ (*bottom left*, *red*), and 879 (36.4%) δ (*bottom right*, *blue*). The number of patients that are deceased at that time point are shown in *gray*.

**Figure 3.**
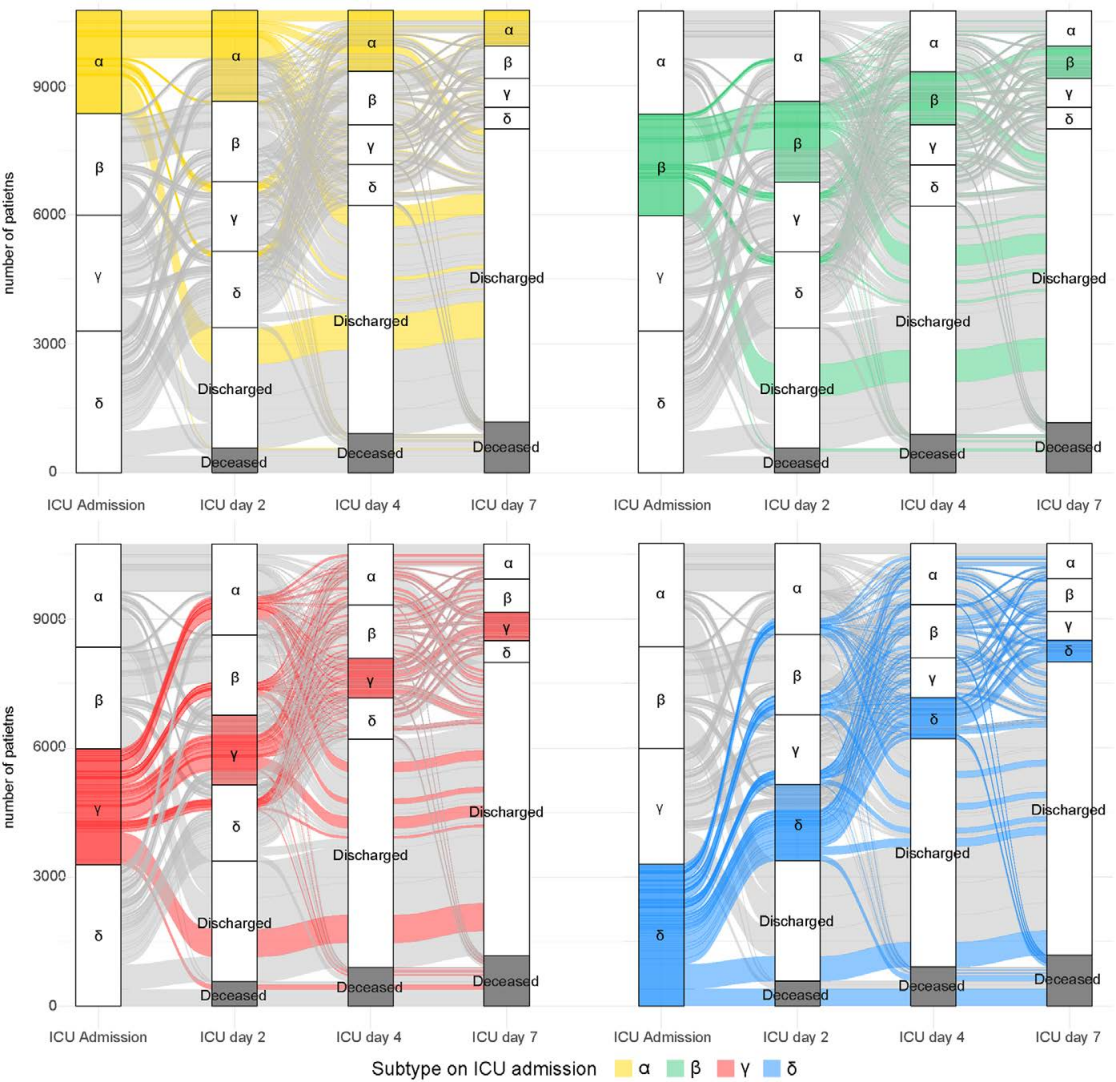
Distribution of subtypes in the Medical Information Mart for Intensive Care-IV (MIMIC-IV) cohort. Distribution of subtypes displayed on ICU admission, days 2, 4, and 7 in the MIMIC-IV cohort. Subtype distribution in sepsis patients admitted to the ICU displayed per subtype at admission in the MIMIC-IV cohort. At admission, 2398 patients (22.3%) has the α subtype (*top left*, *yellow*), 2365 patients (22.0%) β (*top right*, *green*), 3296 (30.8%) γ (*bottom left*, *red*), and 2686 (25.0%) δ (*bottom right*, *blue*). The number of patients that are deceased at that time point are shown in *gray*.

At admission, 6% (*n* = 150) of the admissions were α-type in MARS, 3% (*n* = 70) β-type, 55% (*n* = 1317) γ-type, and 36% (*n* = 879) δ-type (**Fig. 2**). Subtypes in MIMIC-IV were more equally distributed (α = 22% [*n* = 2398], β = 22% [*n* = 2365], γ = 31% [*n* = 3296], and δ = 25% [2686]) (**Fig. 3**).

In MARS, on day 2, the distribution of subtypes in admissions still on the ICU (*n* = 1759) was comparable to ICU admission except for an increase in β (α = 7% [*n* = 130], β = 6% [*n* = 99], γ = 55% [*n* = 975], and δ = 32% [*n* = 555]). Overall in MARS, 40% (709/1759 admissions on day 2) changed subtype between ICU admission and day 2, 45% (518/1133 still admitted on day 4) changed subtype between ICU admission and day 4, and 50% (355/713) between ICU admission and day 7 (Fig. 2). Subtype change from admission to day 2 was most common in the β-type (71% transition) compared with the other subtypes (56% in α-type, 32% in γ-type, and 48% in δ-type) (**Supplementary Table 2**, http://links.lww.com/CCX/B432). For the days thereafter, subtype specific transition was between 35% and 73%. Subtype transition was the least common in the γ-type for any of the time points (32–38%) (Supplementary Table 2, http://links.lww.com/CCX/B432). On day 4, among patients still in the ICU (*n* = 1133), 33% remained in the same subtype as on ICU admission, days 2 and 4. Overall, the distribution of subtypes among patients still admitted to the ICU remained similar on day 4 (*n* = 1133; α = 8%, β = 7%, γ = 57%, and δ = 27%) and day 7 (*n* = 713; α = 9%, β = 7%, γ = 58%, and δ = 26%).

In MIMIC-IV, 43% (3206/7371 patients still admitted on day 2) changed subtype between ICU admission and day 2, 44% (1991/4538 patients still admitted on day 4) changed subtype between ICU admission and day 4, and 47% (1255/2748) between ICU admission and day 7 (Fig. 3). Subtype change was most common in γ-type (ICU admission-day 2: 57% and ICU admission-day 4: 65%) and δ-type (ICU admission-day 4: 65% and ICU admission-day 7: 69%) and the least common in the α-type (28–36%, on all time points) (**Supplementary Table 3**, http://links.lww.com/CCX/B432). On day 4, among admissions still on the ICU (*n* = 4538), 36% remained in the same subtype as on ICU admission, days 2 and 4. On day 2, days 4 and 7 the distribution of subtypes in admissions still on the ICU remained stable (Fig. 3).

It was determined that not a single variable was responsible for switching, but rather a combination of variables and that the hepatic markers aspartate transaminase (AST) and alanine transaminase (ALT) were the most important markers dictating a switch (**Supplementary Table 4**, http://links.lww.com/CCX/B432). According to the effect size, four to 13 variables were significantly responsible for switching subtype from day 0 to day 2 in MARS and four to six in MIMIC-IV, dependent on subtype. Both in MARS and MIMIC-IV, AST and ALT were the most important variables in patients switching from γ and δ on day 0 to another subtype on day 2. This was similar on day 2 to day 4 (Supplementary Table 4, http://links.lww.com/CCX/B432).

### Association of Trajectory With Outcome

The prespecified subgroups of interest, γ-γ (MARS *n* = 648, MIMIC-IV *n* = 834), γ-δ (*n* = 183, *n* = 268), δ-γ (*n* = 270, *n* = 331), and δ-δ (*n* = 347, *n* = 1178) on ICU admission and day 2, did not significantly differ regarding age or sex in MARS (**Table 1**), while a significant difference in age between the δ-δ and δ-γ (63 [50–74] vs. 66 [54–77]; *p* < 0.001) was seen in MIMIC-IV (**Supplementary Table 5**, http://links.lww.com/CCX/B432). Patient admissions with δ-δ and γ-δ had significantly higher SOFA and Acute Physiology and Chronic Health Evaluation scores at admission compared γ-γ and δ-γ patient admissions in both MARS and MIMIC-IV (Table 1; and Supplementary Table 5, http://links.lww.com/CCX/B432).

**TABLE 1.** Comparing Baseline Characteristics and Outcome in Patients With Consistent Subtype on ICU Admission Versus Those Exhibiting a Changed Subtype by Day 2 in the Molecular Diagnosis and Risk Stratification of Sepsis Cohort

Subtype	γ-γ	γ-δ	*p*	δ-δ	δ-γ	*p*
Number of patients	**648**	**183**		**347**	**270**	
Age, median (IQR)	63 (52–71)	64 (54–72)	0.305	64 (53–73)	63 (51–73)	0.426
Sex, male (%)	368 (56.8)	109 (59.6)	0.558	224 (64.6)	175 (64.8)	1.000
Race, White (%)	576 (88.9)	162 (88.5)	0.996	306 (88.2)	235 (87.0)	0.759
Charlson comorbidity score, median (IQR)	1 (0–2)	1 (0–2)	0.491	1 (0–3)	1 (0–2)	0.210
Disease severity at admission						
Sequential Organ Failure Assessment, median (IQR)	6 (4–8)	8 (6–10)	< 0.001	10 (7–12)	8 (56–10)	< 0.001
Acute Physiology and Chronic Health Evaluation IV score, median (IQR)	75 (61–89)	85 (67–103)	< 0.001	102 (79–119)	83 (65–102)	< 0.001
Mechanical ventilation (%)	538 (83.0)	143 (78.1)	0.159	299 (86.2)	222 (82.2)	0.219
Outcome						
Length of stay ICU, median (IQR)	5.00 (3–10)	5 (3–14)	0.281	6 (3–13)	5 (3–11)	0.268
ICU mortality (%)	75 (11.6)	42 (23.0)	< 0.001	120 (34.7)	37 (13.7)	< 0.001
Hospital mortality (%)	132 (20.5)	66 (36.1)	< 0.001	163 (47.1)	64 (23.7)	< 0.001
30-d mortality (%)	109 (17.2)	61 (33.7)	< 0.001	137 (39.6)	62 (23.4)	< 0.001
1-yr mortality (%)	244 (39.4)	94 (52.8)	0.002	199 (58.2)	111 (43.0)	< 0.001

IQR = interquartile range.

Only patients with the δ and γ subtypes were included in this table, since the α and β subtypes are only present in a small proportion of this cohort (on baseline α = 6.2% and β = 2.9%).

**Figure 4.**
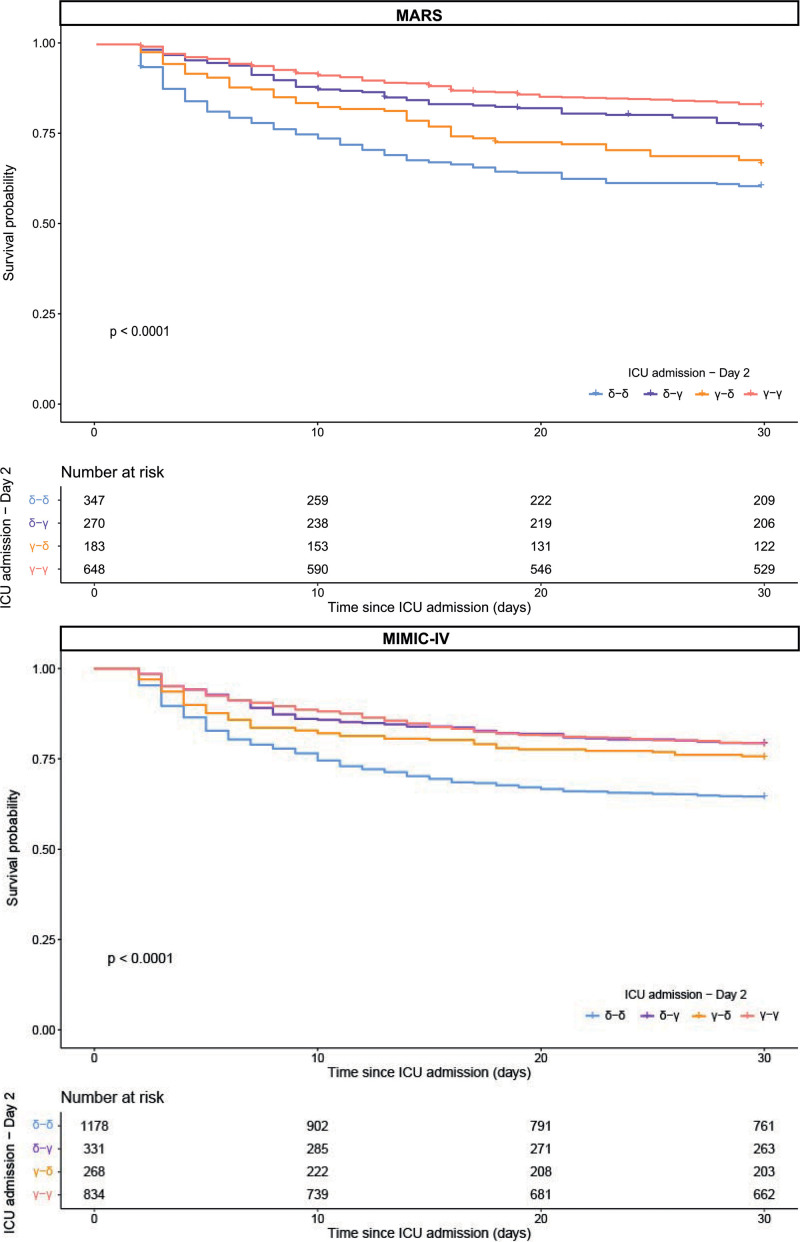
Thirty-day survival curves. The 30-d survival curves are displayed according to the subtypes on ICU admission and day 2 in the Molecular diAgnosis and Risk stratification of Sepsis (MARS) and Medical Information Mart for Intensive Care-IV (MIMIC-IV) cohort. Only patients with the δ and γ subtypes were included in this figure, since the α and γ subtypes were only present in a small proportion of the MARS (on baseline α = 6.2% and β = 2.9%). The logistic regression model between the survival curves of the patients with consistent subtype on ICU admission vs. those exhibiting a changed subtype by day 2 is shown. The model showed significant differences in the MARS between γ-γ and γ-δ (***), δ-δ (***) and δ-γ (*) between δ-γ and δ-δ (***) and γ-δ (*). The model showed significant differences in the MIMIC-IV cohort between δ-δ and γ-γ (***), γ-δ (***) and δ-γ (***). *p < 0.05, **p < 0.01, and ***p < 0.001. *p < 0.05, **p < 0.01, and ***p < 0.001.

Since proportional hazards for a Cox regression were not met, an unadjusted logistic regression was used when analyzing outcome. In MARS, ICU, hospital, 30-day, and 1-year mortality was significantly higher in γ-δ compared with γ-γ (30-d mortality; 34% vs. 17%; *p* < 0.001) and in δ-δ vs. δ-γ (40% vs. 23%; *p* < 0.001). δ-γ was associated with significantly lower 30-day mortality compared with the δ-δ group (odds ratio [OR], 0.46; 95% CI, 0.34–0.61; *p* = 0.010; **Fig. 4**). In contrast, γ-δ was associated with higher 30-day mortality compared with the γ-γ group (OR, 2.47; 95% CI, 1.10–31.97; *p* < 0.001). In MIMIC-IV similar results were found when comparing δ-δ vs. δ-γ (Fig. 4; OR, 0.47; 95% CI, 0.36–0.60; *p* < 0.001). However, when comparing γ-δ vs. γ-γ, the results from the MARS were not repeated in MIMIC-IV (OR, 0.59; 95% CI, 0.45–0.76; *p* = 0.210).

In the first sensitivity analysis, 30-day mortality was compared using an unadjusted logistic regression in α-α to α-γ and α-δ and in β-β to β-γ and β-δ. In MARS, no significantly different results were found; however, all groups were fairly small (ranging from 11 to 41 patients per group; **Supplementary Fig. 5**, http://links.lww.com/CCX/B432). In MIMIC-IV, α-α was associated with a significant lower 30-day mortality compared with α-γ and α-δ (OR, 0.50; 95% CI, 0.35–0.72 and OR, 0.30; 95% CI, 0.20–0.47, respectively). β-β was associated with a significant lower 30-day mortality compared with β-δ (OR, 0.48; 95% CI, 0.36–0.63; Supplementary Fig. 5, http://links.lww.com/CCX/B432), comparing β-β to β-γ did not result into significantly different outcomes.

In the second sensitivity analysis, 30-day mortality was also compared in patients grouped into subtype at admission-day 4 and at admission-day 7 in patients with δ-γ vs. δ-δ and γ-δ vs. γ-γ. Similar results as when comparing admission-day 2 were found. In MARS, when looking into admission-day 4 δ-γ was associated with significantly lower 30-day mortality compared with the δ-δ group (OR, 0.56; 95% CI, 0.39–0.81) and γ-δ was associated with higher 30-day mortality compared with the γ-γ group (OR, 2.63; 95% CI, 1.75–3.93; **Supplementary Fig. 6**, http://links.lww.com/CCX/B432). In MIMIC-IV similar results were found when comparing δ-γ vs. δ-δ (OR, 0.61; 95% CI, 0.46–0.81) and γ-δ vs. γ-γ (OR, 1.75; 95% CI, 1.23–2.48; Supplementary Fig. 6, http://links.lww.com/CCX/B432). Similar results were found when looking into admission-day 7 (in MARS: δ-γ vs. δ-δ [OR, 0.39; 95% CI, 0.25–0.61] and γ-δ vs. γ-γ [OR, 3.11; 95% CI, 1.89–5.09] and in MIMIC-IV: δ-γ vs. δ-δ [OR, 0.44; 95% CI, 0.31–0.60] and γ-δ vs. γ-γ [OR, 2.13; 95% CI, 1.36–3.33]; **Supplementary Fig. 7**, http://links.lww.com/CCX/B432).

### Association of Trajectory With Host Response Biomarkers

**Figure 5.**
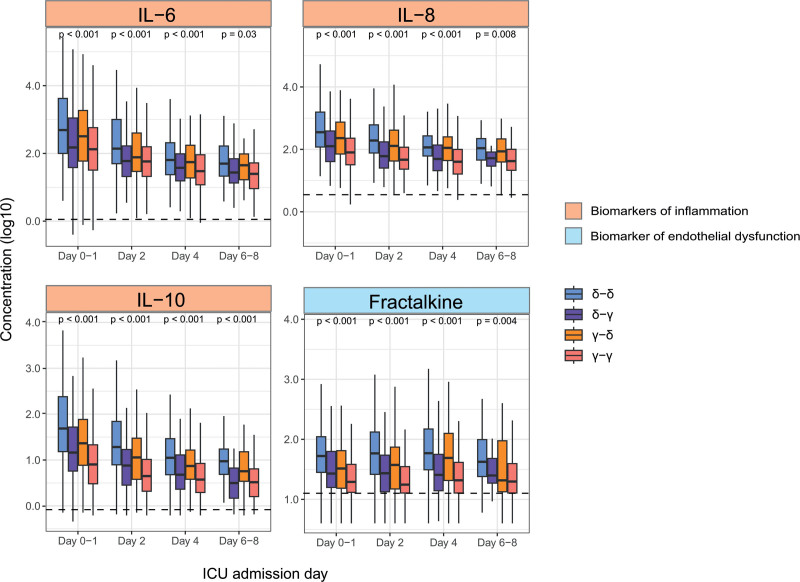
Host response biomarkers. Interleukin (IL)-6, IL-8, IL-10, and fractalkine during ICU admission are displayed according to the subtypes on ICU admission and day 2 in the MARS cohort. *Boxplots* displaying biomarker concentrations (pg/mL), after log10 transformation. *Horizontal line* is the median concentration of healthy volunteers (*n* = 25). For the complete overview of all measured biomarkers, see Supplementary Figure 8 (http://links.lww.com/CCX/B432).

Of 12 biomarkers, significant differences were present for most markers (excluding E-selectin [biomarker of endothelial dysfunction]) on day 2 and day 4 (**Supplementary Fig. 8** and **Supplementary Table 6**, http://links.lww.com/CCX/B432). The most aberrant biomarkers were seen in the δ-δ group, followed by the γ-δ group, independent of the day or biomarker domain (inflammation, coagulation, or endothelial dysfunction). After adjustment for multiple comparisons, seven biomarkers (four biomarkers of inflammation: IL-6, IL-8, IL-10, and MMP-8 and three biomarkers of endothelial dysfunction: fractalkine, angiopoietin-2/angiopoietin-1, and angiopoietin-2) in the δ-δ group at baseline were significantly more aberrant compared with the γ-δ group. In addition, four biomarkers (IL-6, IL-8, IL-10, and fractalkine) were significantly more aberrant in the δ-δ group at baseline compared with the δ-γ group, suggesting a potential prediction of baseline biomarker to subtype change (**Fig. 5**). The longitudinal change of almost all biomarkers did not differ between groups in a linear mixed model. Only IL-8, IL-10, and protein C (biomarker of coagulation) showed a different trajectory in the γ-γ group compared with δ-δ (**Supplementary Table 7**, http://links.lww.com/CCX/B432).

## DISCUSSION

This study demonstrates that previously established sepsis subtypes are dynamic over time, prognostic of outcome and associated with host response alterations. Sepsis subtype prevalence remained relatively stable throughout the first week of ICU admission, yet individual patients quite often transitioned between subtypes over time. Patients remaining or transitioning to subtype δ were associated with higher mortality rates compared with patients remaining or switching to the other subtypes, regardless of the subtype they originated from. Longitudinal host response biomarkers reflecting inflammation, coagulation, and endothelial dysfunction were more aberrant in patients remaining or switching to δ compared with γ in the first 2 days after ICU admission.

In this study, subtypes were relatively unstable since approximately 40% of patients had transitioned to a different subtype on the following assessment day. This instability parallels findings from an analysis in COVID-19 patients, introducing the concept of phenotype half-life (18). Phenotype half-life is the time that a subtype has lost half of its allocated patients. In our study, the half-life was 3–4 days compared with 3–6 days reported in COVID-19 (18). The relative short half-life indicates the importance of understanding longitudinal trajectories and their relation to treatment and outcome. While longitudinal data provides more comprehensive insights compared with cross-sectional data, the real benefit lays within the ability to predict longitudinal changes before the occurrence of real time changes. In this study, at baseline, discernible differences in clinical characteristics and biomarkers were already evident between patients who changed subtypes and those who remained within the same subtype. As such, patients transitioning from γ-δ show a more similar biomarker pattern at admission to δ-δ patients compared with patients in γ on day 2 (both δ-γ and γ-γ) This suggests that certain variables, such as plasma protein biomarkers, may serve as early indicators of an impending change in subtype. The ability to predict an upcoming change would be a potentially promising method for prognostication. For instance, in a prospective observational study examining the trajectories of host response profiles in severe COVID-19, patients progressing from noninvasive respiratory support to invasive mechanical ventilation exhibited significantly higher baseline levels of soluble receptor for advanced glycation end products, indicating a potential avenue for early intervention and personalized treatment strategies (19).

Additionally, our study highlights differences in mortality based on subtype trajectories, aligning with descriptions of these subtypes in the original study (8): patients with subtype δ presented with liver dysfunction, septic shock, and higher mortality, while those with subtype γ showed more inflammation, pulmonary dysfunction, and lower mortality compared with δ. When the clinical condition (and thus variables) of a patient worsens, a shift toward the δ subtype is expected. The prognostic nature of subtype trajectories, rather than single time point assessed subtypes, has been previously established. Using group-based trajectory modeling of repeated temperature measurements, four sepsis subtypes were identified: hyperthermic, slow resolvers (mortality 10%); hyperthermic, fast resolvers (mortality 3%); normothermic (mortality 5%); and hypothermic (mortality 9%) (9). Furthermore, vital sign (10) and organ dysfunction trajectories (11) in the first hours to days (8–72 hr) have been used to phenotype sepsis patients both associated with distinct outcomes. Furthermore, different responses to treatment with balanced crystalloids vs. saline were found (10, 20). Together, this emphasizes the importance of tracking individual subtype trajectories for better prognostic and predictive enrichment in sepsis patients.

The high mortality rates in sepsis patients have underscored the imperative to focus on a more tailor-made approach (21). However, the clinical variables or biomarkers used must then be responsive to treatment and reflective of underlying pathology. Designing personalized trials based on ICU admission subtypes may face challenges if patients transition to other subtypes after inclusion. The inclusion window of clinical trials is mostly 48 hours, and our results show that after 2 days of ICU admission approximately 40% of the patients changed subtype, indicating a possible limited target population for personalized treatment. Many researchers advocate for gaining deeper insights into the longitudinal dynamics of the host response and hemodynamics in sepsis (7, 12, 22). However, to the best of our knowledge, integrating longitudinal subtypes in clinical trials in sepsis patients has not yet been executed.

Strengths of this study include extensive and reliable daily clinical data collection during ICU admission, repeated measurements of protein plasma biomarkers, and the utilization of two distinct cohorts. However, several limitations should be acknowledged. First, the influence of treatment during ICU stay might have influenced the changes in subtypes over time. Nevertheless, it is plausible that remaining in, or transitioning between, subtypes could correlate with outcome regardless of treatment effects. Second, due to the difference in the availability of the daily clinical variables in both MARS and MIMIC-IV, the variables used for subtype adjudication slightly differed, also in relation to the original analysis (8). This could have influenced subtype membership and trajectories. Third, the reason for the frequent transitioning between subtypes during the first week of ICU could not be answered with complete certainty due to the observational design of this study. Last, also because of the observational design, any firm conclusions on casual relationships between subtype trajectories and outcomes cannot be drawn.

## CONCLUSIONS

In conclusion, this study illuminates the dynamic nature of previously established sepsis subtypes, their prognostic significance, and their association with host response changes. Over the first week of ICU admission, patients frequently transitioned between subtypes during their stay. Patients remaining in or transitioning to subtype δ exhibited higher mortality rates compared with those in other subtypes. Furthermore, longitudinal host response biomarkers reflecting inflammation, coagulation, and endothelial dysfunction were more disrupted in patients remaining in or transitioning to subtype δ compared with γ, even though, trajectories were similar. These findings underscore the importance of monitoring sepsis subtypes and their associated host responses for improved prognostication and personalized treatment strategies.

## Supplementary Material
