# Supplementary material for: Clinical Subtype Trajectories in Sepsis Patients Admitted to the ICU: A Secondary Analysis of an Observational Study
Source: Crit Care Explor. 2024 Nov 14;6(11):e1176. doi: 10.1097/CCE.0000000000001176 (PMC11567702; doi:10.1097/CCE.0000000000001176)
Supplement: Supplementary file 1 [file cc9-6-e1176-s001.pdf]

Online Supplement

Clinical subtype trajectories in sepsis patients admitted to the intensive care unit: a secondary analysis of an observational study

Marleen A. Slim*, MD, Rombout B.E. van Amstel*, MD, Marcella. C.A. Müller, MD, PhD, Olaf L. Cremer, MD, PhD, Alexander. P.J. Vlaar, MD, PhD, MARS Consortium, Tom van der Poll, MD, PhD, W. Joost Wiersinga, MD, PhD, Christopher W. Seymour, MD, MSc, Lonneke A. van Vught, MD, PhD

**Both authors contributed equally.*

**Content**	**Page**
Supplementary methods	2
Supplementary results	3
Supplementary Table 1. Baseline characteristics and outcomes of patients admitted to the ICU	4
Supplementary Table 2. Overview of patients remaining in or transitioning to subtype on ICU admission, day 2, 4 and 7 in the MARS cohort	5
Supplementary Table 3. Overview of patients remaining in or transitioning to subtype on ICU admission, day 2, 4 and 7 in the MIMIC-IV cohort	6
Supplementary Table 4. Assessment of which variables determine if a patient switches subtypes or stays within the same subtype	7
Supplementary Table 5. Comparing baseline characteristics and outcome in patients with consistent subtype on ICU admission versus those exhibiting a changed subtype by day 2 in the MIMIC-IV cohort	8
Supplementary Table 6. Absolute biomarker concentrations stratified per subtype on ICU admission-day 2 and per day	9
Supplementary Table 7. Longitudinal change in the biomarkers in a linear mixed model compared to δ-δ	11
Supplementary Figure 1. Percentage of the missing variables on ICU admission in the MARS cohort	12
Supplementary Figure 2. Percentage of the missing variables on day 2, 4 and 7 in the MARS cohort	13
Supplementary Figure 3. Percentage of the missing variables on ICU admission in the MIMIC-IV cohort	14
Supplementary Figure 4. Percentage of the missing variables on day 2, 4, and 7 in the MIMIC-IV cohort	15
Supplementary Figure 5. 30-day survival curves according to the subtypes on ICU admission and day 2 in patients with subtype α or β on admission	16
Supplementary Figure 6. 30-day survival curves according to the subtypes on ICU admission and day 4	17
Supplementary Figure 7. 30-day survival curves according to the subtypes on ICU admission and day 7	18
Supplementary Figure 8. Host response biomarkers	19

Supplementary methods

Patients

Within the MARS, information on demographics, daily clinical, laboratory and outcome data and severity indices such as Acute Physiology and Chronic Health Evaluation (APACHE) IV[1] and Sequential Organ Failure Assessment (SOFA) scores[2] were prospectively collected by trained and dedicated ICU researchers. For every patient included in the MARS cohort, the plausibility of an infection was assessed retrospectively using a four-point scale (ascending from none, possible, probable to definite) using the Centers for Disease Control and Prevention[3] and International Sepsis Forum consensus definitions[4] as described[5]. In this secondary analyses all patients fulfilling the Sepsis-3[2], criteria were included defined as any suspected infection for which the clinical team initiated antibiotics, within 48 hours after ICU admission, accompanied by organ failure as indicated by a SOFA score[2] of two or more (central nervous system excluded). When the SOFA score was not available the following variables were used to define organ failure: mechanical ventilation, acute kidney injury (AKI), acute respiratory distress syndrome (ARDS) or shock, all on the first day of ICU admission. AKI and ARDS were defined using strict pre-set criteria[6,7]. Shock was defined as the use of noradrenaline for hypotension in a dose of >0.1 µg/kg/min during at least 50% of the day.

Adjudication to clinical subtypes

Since the Elixhauser comorbidity index was not available in either cohort, the Charlson comorbidity index[8] was calculated. When the international normalized ratio (INR) was missing in the MARS cohort, the prothrombin time (PT) value was used to calculate the INR.

Assessment of which variables determine if a patient switches subtypes or stays within the same subtype

To assess which variables determined if a patient switches subtypes or stays within the same subtype, the following analyses were executed: first, for each patient, the difference in Euclidean Distance for various variables between specific time points was calculated. For instance, the Euclidean Distance for heart rate was measured on day 0 and day 2, and the difference between these values was calculated. Next, it was assessed whether these differences were significantly different between patients who remained in the same subtype and those who switched to another subtype. To do this, the effect size using Hedges' G was calculated [9]. For easier interpretation, the effect sizes were categorized based on Cohen's guidelines [10]: less than 0.2 = negligible, between 0.2 and 0.5 = small, between 0.5 and 0.8 = moderate, and greater than 0.8 = large.

Biomarker measurements

Whole blood samples were centrifuged directly (1500 G for 15 min) and frozen at -80°C within 4 hours after blood draw. Interleukin (IL)-6, IL-8, IL-10, soluble intercellular adhesion molecule (ICAM)-1, soluble E-selectin and fractalkine were measured by FlexSet cytometric bead array (BD Biosciences, San Jose, CA) using FACS Calibur (Becton Dickenson, Franklin Lakes, NJ). Matrix metalloproteinase (MMP)-8, angiopoietin-1, angiopoietin-2 and protein C (all R&D systems, Abingdon, UK) and D-dimer (Procartaplex, eBioscience, San Diego, CA) were measured by Luminex multiplex assay using BioPlex 200 (BioRad, Hercules, CA).

Supplementary results

Subtype trajectories

The erythrocyte sedimentation rate (ESR), total number of bands and Glasgow Coma Scale (GCS) were unavailable daily in MARS and thus excluded. No clinical variables exceeded >80% missing on ICU admission (Supplementary Figure 1). In MIMIC-IV, C-reactive protein (CRP) and ESR had >80% missing on baseline and were therefore excluded (Supplementary Figure 3).

Supplementary Table 1. Baseline characteristics and outcomes of patients admitted to the ICU

**Cohort**	**MARS cohort**	**MIMIC-IV cohort**
**Number of patients**	**2416**	**10745**
Age (median [IQR])	63 [51, 72]	67 [55, 79]
Sex = male (%)	1484 (61.4)	6201 (57.7)
Race = White (%)	2116 (87.6)	7044 (65.6)
Charlson comorbidity score (median [IQR])	1 [0, 2]	3 [1, 4]
**Disease severity on admission**		
SOFA (median [IQR])	7 [4, 9]	3 [2, 5]
APACHE III Score (median [IQR])*		53 [39, 75]
APACHE IV Score (median [IQR])**	76 [59, 97]	
Mechanical ventilation (%)	1813 (75.0)	2585 (24.1)
**Vital signs and laboratory values on admission**		
Respiratory rate (median [IQR])	33 [27, 40]	28 [24, 32]
Heart rate (median [IQR])	128 [113, 146]	106 [92, 121]
Systolic blood pressure (median [IQR])	80 [71, 91]	87 [78, 97]
CRP mg/L (median [IQR])	122 [44, 235]	93 [37, 174]
White cell count *10^9/L (median [IQR])	13.85 [9.10, 19.40]	14.10 [9.88, 19.50]
Platelets *10^9/L (median [IQR])	182 [115, 266]	153 [102, 220]
Creatinine µmol/L(median [IQR])	104 [71, 171]	106 [80, 177]
**Outcome**		
LOS ICU (median [IQR])	3 [1, 8]	3 [1, 7]
ICU mortality (%)	440 (18.2)	1632 (15.2)
Hospital mortality (%)	681 (28.2)	2208 (20.5)
30-day mortality (%)	619 (26.0)	2174 (20.2)
1-year mortality (%)	1052 (44.9)	2536 (23.6)

*Range: 0-299. **Range: 0-286. Abbreviations: APACHE, acute physiology and chronic health evaluation; ICU, intensive care unit; SOFA, sequential organ failure assessment

Supplementary Table 2. Overview of patients remaining in or transitioning to subtype on ICU admission, day 2, 4 and 7 in the MARS cohort

ICU admission – day 2				
**Subtype on ICU admission**	**Remained in same subtype on day 2**	**Transitioned to different subtype on day 2**	**Discharged alive on day 2**	**Deceased on day 2**
α (n=150)	41 (27%* & 44%†)	52 (35%* & 56%†)	55 (37%)	2 (1%)
β (n=70)	14 (20%* & 29%†)	35 (50%* & 71%†)	21 (30%)	0 (0%)
γ (n=1317)	648 (49%* & 68%†)	302 (23%* & 32%†)	340 (26%)	27 (2%)
δ (n=879)	347 (39%* & 52%†)	320 (36%* & 48%†)	110 (13%)	102 (12%)
**Day 2 – day 4**				
**Subtype on day 2**	**Remained in same subtype on day 4**	**Transitioned to different subtype on day 4**	**Discharged alive on day 4**	**Deceased on day 4**
α (n=130)	31 (24%* & 45%†)	38 (29%* & 55%†)	57 (44%)	4 (3%)
β (n=99)	17 (17%* & 35%†)	32 (32%* & 65%†)	47 (47%)	3 (3%)
γ (n=975)	441 (45%* & 69%†)	202 (21%* & 34%†)	302 (31%)	30 (3%)
δ (n=555)	185 (33%* & 50%†)	187 (34%* & 52%†)	124 (22%)	59 (11%)
**Day 4 – day 7**				
**Subtype on day 4**	**Remained in same subtype on day 7**	**Transitioned to different subtype on day 7**	**Discharged alive on day 7**	**Deceased on day 7**
α (n=95)	20 (21%* & 39%†)	31 (33%* & 73%†)	41 (43%)	3 (3%)
β (n=82)	16 (20%* & 36%†)	28 (34%* & 61%†)	37 (45%)	1 (1%)
γ (n=645)	275 (43%* & 66%†)	144 (22%* & 35%†)	205 (32%)	21 (3%)
δ (n=311)	95 (31%* & 48%†)	104 (33%* & 57%†)	83 (27%)	29 (9%)
**ICU admission – day 4**				
**Subtype on ICU admission**	**Remained in same subtype on day 4**	**Transitioned to different subtype on day 4**	**Discharged alive on day 4**	**Deceased on day 4**
α (n=150)	14 (9%* & 27%†)	38 (25%* & 73%†)	90 (60%)	8 57%)
β (n=70)	9 (13%* & 39%†)	14 (20%* & 61%†)	47 (67%)	0 (0%)
γ (n=1317)	399 (30%* & 65%†)	214 (16%* & 35%†)	646 (49%)	58 (4%)
δ (n=879)	193 (22%* & 43%†)	252 (29%* & 57%†)	273 (31%)	161 (18%)
**ICU admission – day 7**				
**Subtype on ICU admission**	**Remained in same subtype on day 7**	**Transitioned to different subtype on day 7**	**Discharged alive on day 7**	**Deceased on day 7**
α (n=150)	15 (10%* & 41%†)	22 (15%* & 59%†)	102 (68%)	11 (7%)
β (n=70)	5 (7%* & 36%†)	9 (13%* & 64%†)	55 (79%)	1 (1%)
γ (n=1317)	226 (17%* & 62%†)	136 (10%* & 38%†)	875 (66%)	80 (6%)
δ (n=879)	112 (13%* & 37%†)	188 (21%* & 63%†)	390 (44%)	189 (22%)

Abbreviations: ICU, intensive care unit, * percentage of all admissions on ICU admission, † percentage of admissions still on the ICU on the corresponding admission day

Supplementary Table 3. Overview of patients remaining in or transitioning to subtype on ICU admission, day 2, 4 and 7 in the MIMIC-IV cohort

		**ICU admission – day 2**		
**Subtype on ICU admission**	**Remained in same subtype on day 2**	**Transitioned to different subtype on day 2**	**Discharged alive on day 2**	**Deceased on day 2**
α (n=2398)	1100 (46%* & 72%†)	431 (18%* & 28%†)	843 (35%)	24 (1%)
β (n=2365)	1053 (45%* & 67%†)	516 (22%* & 33%†)	745 (32%)	51 (2%)
γ (n=2686)	834 (31%* & 43%†)	1104 (41%* & 57%†)	639 (24%)	109 (4%)
δ (n=3296)	1178 (36%* & 50%†)	1155 (35%* & 50%†)	572 (17%)	391 (12%)
		**Day 2 – day 4**		
**Subtype on day 2**	**Remained in same subtype on day 4**	**Transitioned to different subtype on day 4**	**Discharged alive on day 4**	**Deceased on day 4**
α (n=2110)	811 (38%* & 68%†)	374 (18%* & 32%†)	904 (43%)	21 (1%)
β (n=1872)	722 (39%* & 67%†)	357 (19%* & 33%†)	741 (40%)	52 (3%)
γ (n=1619)	402 (25%* & 40%†)	597 (37%* & 60%†)	546 (34%)	74 (5%)
δ (n=1770)	612 (35%* & 48%†)	663 (37%* & 52%†)	313 (18%)	182 (10%)
		**Day 4 – day 7**		
**Subtype on day 4**	**Remained in same subtype on day 7**	**Transitioned to different subtype on day 7**	**Discharged alive on day 7**	**Deceased on day 7**
α (n=1415)	525 (37%* & 64%†)	301 (21%* & 36%†)	557 (39%)	32 (2%)
β (n=1237)	434 (35%* & 64%†)	249 (20%* & 36%†)	491 (40%)	63 (5%)
γ (n=929)	256 (28%* & 43%†)	337 (36%* & 57%†)	275 (30%)	61 (7%)
δ (n=957)	278 (29%* & 43%†)	368 (38%* & 57%†)	196 (20%)	115 (12%)
		**ICU admission – day 4**		
**Subtype on ICU admission**	**Remained in same subtype on day 4**	**Transitioned to different subtype on day 4**	**Discharged alive on day 4**	**Deceased on day 4**
α (n=2398)	627 (26%* & 70%†)	267 (11%* & 30%†)	1454 (61%)	50 (2%)
β (n=2365)	558 (24%* & 63%†)	322 (14%* & 37%†)	1378 (58%)	107 (7%)
γ (n=2686)	417 (16%* & 35%†)	762 (28%* & 65%†)	1316 (49%)	191 (7%)
δ (n=3296)	604 (18%* & 38%†)	981 (30%* & 62%†)	1155 (35%)	556 (17%)
		**ICU admission – day 7**		
**Subtype on ICU admission**	**Remained in same subtype on day 7**	**Transitioned to different subtype on day 7**	**Discharged alive on day 7**	**Deceased on day 7**
α (n=2398)	363 (15%* & 66%†)	186 (8%* & 34%†)	1770 (74%)	79 (3%)
β (n=2365)	285 (12%* & 62%†)	178 (8%* & 38%†)	1742 (74%)	160 (7%)
γ (n=2686)	260 (10%* & 67%†)	451 (17%* & 63%†)	1731 (64%)	244 (9%)
δ (n=3296)	317 (10%* & 31%†)	708 (21%* & 69%†)	1579 (48%)	692 (21%)

Abbreviations: ICU, intensive care unit, * percentage of all admissions on ICU admission, † percentage of admissions still on the ICU on the corresponding admission day

Supplementary Table 4. Assessment of which variables determine if a patient switches subtypes or stays within the same subtype

Subtype transitioning	Variable 1	Effect size	Variable 2	Effect size	Variable 3	Effect size	# of variables *
**MARS - Day 0-2**							
**α to other**	Albumin	-0.68; Moderate	WBC	-0.51; Moderate	Troponin	-0.49; Small	13
**β to other**	Heart rate	0.59; Moderate	Troponin	0.53; Moderate	AST	-0.54; Moderate	13
**γ to other**	ALT	-0.66; Moderate	AST	-0.64; Moderate	Troponin	-0.27; Small	6
**δ to other**	Troponin	-0.46; Small	ASAT	-0.35; Small	ALT	-0.35; Small	4
**MIMIC-IV – Day 0-2**							
**α to other**	Albumin	-0.41; Small	AST	-0.33; Small	ALT	-0.29; Small	5
**β to other**	AST	-0.52; Moderate	ALT	-0.46; Small	Urea	-0.30; Small	5
**γ to other**	AST	-0.45; Small	ALT	-0.44; Small	Bands	-0.36; Small	4
**δ to other**	AST	-0.36; Small	Bilirubin	-0.31; Small	ALT	-0.30; Small	6
**MARS - Day 2-4**							
**α to other**	Troponin	-0.54; Moderate	Creatinin	-0.48; Small	Respirato-ry rate	-0.43; Small	9
**β to other**	Respirato-ry rate	-0.86; Large	ALT	-0.62; Moderate	Lactate	-0.58; Moderate	18
**γ to other**	AST	-0.58; Moderate	ALT	-0.56; Moderate	Troponin	-0.35; Small	6
**δ to other**	AST	-0.46; Small	Troponin	-0.40; Small	ALT	-0.40; Small	8
**MIMIC-IV – Day 2-4**							
**α to other**	Bands	-0.36; Small	Albumin	-0.36; Small	Respirato-ry rate	-0.27; Small	3
**β to other**	AST	-0.51; Small	ALT	-0.47; Small	Urea	-0.27; Small	5
**γ to other**	AST	-0.41; Small	Bands	-0.36; Small	ALT	-0.34; Small	4
**δ to other**	ALT	-0.49; Small	AST	-0.45; Small	Bilirubin	-0.44; Small	6

* Number of variables that have significant effect sizes, so without the classification 'Negligible'.
Abbreviations: ALT, alanine transaminase; AST, aspartate aminotransferase; WBC, white blood cell count.

Supplementary Table 5. Comparing baseline characteristics and outcome in patients with consistent subtype on ICU admission versus those exhibiting a changed subtype by day 2 in the MIMIC-IV cohort

Subtype	γ-γ	γ-δ	*P*-value	δ-δ	δ-γ	*P*-value
**Number of patients**	**834**	**268**		**1178**	**331**	
Age (median [IQR])	68 [56, 79]	66 [56, 77]	0.217	63 [50, 74]	66 [54, 77]	<0.001
Sex = male (%)	411 (49.3)	149 (55.6)	0.084	732 (62.1)	181 (54.7)	0.017
Race = White (%)	575 (68.9)	181 (67.5)	0.722	706 (59.9)	213 (64.4)	0.164
Charlson comorbidity score (median [IQR])	2.00 [1.00, 3.00]	2.00 [1.00, 4.00]	0.014	3 [2, 5]	2 [1, 4]	<0.001
**Disease severity on admission**						
APACHE III Score (median [IQR])	53 [42, 71]	67 [50, 90]	<0.001	89 [67, 110]	70 [51, 87]	<0.001
SOFA (median [IQR])	3 [2, 4]	4 [2, 5]	0.001	5 [3, 7]	4 [3, 6]	<0.001
Mechanical ventilation (%)	274 (32.9)	83 (31.0)	0.618	318 (27.0)	86 (26.0)	0.766
**Outcome**						
LOS ICU (median [IQR])	4 [3, 8]	6 [3, 12]	<0.001	6 [3, 12]	4 [3, 9]	<0.001
ICU mortality (%)	118 (14.1)	53 (19.8)	0.034	357 (30.3)	49 (14.8)	<0.001
Hospital mortality (%)	172 (20.6)	66 (24.6)	0.194	435 (36.9)	71 (21.5)	<0.001
30-day mortality (%)	175 (21.0)	66 (24.6)	0.242	419 (35.6)	68 (20.5)	<0.001
1-year mortality (%)	209 (25.1)	79 (29.5)	0.176	469 (39.8)	85 (25.7)	<0.001

Only patients with the δ and γ subtype were included in this table, since this was also done for the MARS cohort. Abbreviations: ICU, intensive care unit; LOS, length of stay; SOFA, sequential organ failure assessment

Supplementary Table 6. Absolute biomarker concentrations stratified per subtype on ICU admission-day 2 and per day – part I

	δ-δ	δ-γ	γ-δ	γ-γ	*P*-value
**Day 0-1**					
**n**	202	147	116	384	
**IL-6 (median [IQR])**	489.50 [99.79, 4195.53]	150.07 [38.28, 1110.38]	321.57 [59.39, 1853.53]	132.68 [32.12, 576.49]	<0.001
**IL-8 (median [IQR])**	354.36 [118.91, 1563.83]	126.33 [40.74, 388.54]	229.86 [71.06, 745.60]	79.84 [32.11, 227.55]	<0.001
**IL-10 (median [IQR])**	48.69 [15.25, 240.01]	14.54 [5.74, 52.26]	23.16 [7.68, 76.64]	8.00 [3.04, 21.43]	<0.001
**MMP8 (median [IQR])**	5251.62 [1616.86, 13196.17]	2523.78 [698.71, 6871.61]	4525.14 [1113.33, 11164.65]	2240.77 [731.41, 6708.44]	<0.001
**D-Dimer (median [IQR]) ***	13046.00 [5441.93, 24360.75]	9701.20 [4576.95, 20292.50]	7867.20 [3241.78, 13518.50]	7688.90 [3462.53, 14497.50]	<0.001
**Protein C (median [IQR])**	109079.55 [86365.36, 140780.30]	117332.81 [90693.10, 170142.94]	116477.39 [89541.41, 159345.67]	117888.20 [86560.78, 159519.59]	0.065
**Ang-1 (median [IQR])**	1380.20 [656.18, 3486.00]	2227.59 [957.60, 6758.82]	1535.09 [693.23, 4887.58]	2985.38 [986.31, 7338.64]	<0.001
**Ang-2 (median [IQR])**	12416.80 [5182.49, 23909.17]	6482.24 [2581.20, 14280.36]	8392.02 [4208.86, 20960.35]	5752.30 [3014.58, 10302.76]	<0.001
**Ang-2/-ang-1 (median [IQR])**	7.78 [2.42, 26.20]	2.47 [0.63, 11.60]	6.17 [1.06, 15.67]	1.71 [0.61, 6.15]	<0.001
**E-Selectin (median [IQR])**	36.55 [15.22, 92.88]	35.42 [17.07, 69.49]	45.03 [18.72, 89.44]	28.15 [13.74, 67.69]	0.016
**Fractalkine (median [IQR])**	52.74 [28.19, 110.49]	26.92 [15.80, 62.78]	32.68 [15.33, 64.75]	19.54 [13.08, 38.27]	<0.001
**ICAM (median [IQR])**	212462.43 [116911.62, 366076.91]	159551.04 [94256.15, 269930.75]	232272.22 [145282.72, 337400.02]	159924.58 [97542.81, 262899.30]	<0.001
**Day 2**					
**n**	218	161	121	394	
**IL-6 (median [IQR])**	138.58 [49.85, 1005.63]	59.62 [20.80, 166.36]	76.75 [29.41, 399.49]	57.90 [20.94, 156.92]	<0.001
**IL-8 (median [IQR])**	192.24 [76.49, 609.54]	60.56 [25.20, 173.43]	129.12 [42.47, 412.48]	46.61 [23.25, 117.00]	<0.001
**IL-10 (median [IQR])**	19.19 [7.87, 69.45]	7.58 [2.84, 16.95]	11.39 [3.80, 29.86]	4.47 [2.09, 10.31]	<0.001
**MMP8 (median [IQR])**	4273.32 [1205.04, 15234.74]	1535.56 [516.39, 6034.57]	2712.16 [852.96, 10062.60]	1796.61 [535.68, 4776.36]	<0.001
**D-Dimer (median [IQR]) ***	13319.00 [5805.10, 24732.00]	8341.30 [3618.40, 15877.00]	8875.50 [3932.50, 15760.00]	7634.85 [3710.65, 14444.25]	<0.001
**Protein C (median [IQR])**	106118.10 [79618.55, 136702.69]	120290.20 [94504.16, 159039.47]	114613.73 [93159.49, 145838.58]	131012.66 [97172.38, 169424.87]	<0.001
**Ang-1 (median [IQR])**	1031.27 [545.71, 2186.14]	1472.00 [672.52, 3270.81]	1315.74 [595.23, 2874.56]	2295.20 [911.70, 5430.81]	<0.001
**Ang-2 (median [IQR])**	18405.06 [8342.35, 40078.72]	8047.49 [3561.18, 15246.79]	12010.82 [4185.33, 21861.26]	5595.03 [2748.61, 10487.39]	<0.001
**Ang-2/-ang-1 (median [IQR])**	20.98 [5.44, 50.93]	5.29 [1.49, 16.52]	8.91 [2.08, 31.12]	2.30 [0.67, 9.28]	<0.001
**E-Selectin (median [IQR])**	37.11 [17.70, 79.08]	33.74 [13.81, 65.24]	37.44 [15.84, 82.03]	28.84 [13.48, 54.71]	0.002
**Fractalkine (median [IQR])**	58.30 [26.10, 132.28]	27.24 [13.37, 54.39]	37.44 [14.91, 74.17]	17.59 [12.75, 35.11]	<0.001
**ICAM (median [IQR])**	264604.01 [139960.44, 417702.93]	189431.08 [111981.84, 324731.67]	227402.56 [146374.12, 332676.82]	181294.90 [110650.70, 290225.19]	<0.001

Supplementary Table 6. Absolute biomarker concentrations stratified per subtype on ICU admission-day 2 and per day – part II

	δ-δ	δ-γ	γ-δ	γ-γ	*P*-value
			**Day 4**		
**n**	146	109	85	275	
**IL-6 (median [IQR])**	63.94 [23.62, 206.19]	37.54 [15.51, 97.13]	55.25 [18.87, 173.80]	29.94 [11.84, 91.06]	<0.001
**IL-8 (median [IQR])**	115.73 [61.42, 271.02]	49.33 [20.80, 137.55]	111.74 [43.94, 251.78]	40.10 [16.24, 99.59]	<0.001
**IL-10 (median [IQR])**	11.18 [4.79, 29.22]	4.78 [2.31, 12.90]	7.41 [3.81, 16.49]	3.75 [1.96, 8.42]	<0.001
**MMP8 (median [IQR])**	2776.02 [745.41, 7155.01]	1177.99 [540.21, 3640.92]	2049.03 [683.54, 5724.20]	1275.73 [506.97, 3380.81]	<0.001
**D-Dimer (median [IQR]) ***	13635.00 [5853.43, 23796.75]	9833.90 [5377.20, 17235.00]	8629.00 [4403.70, 16758.00]	9368.00 [5354.75, 16941.00]	0.005
**Protein C (median [IQR])**	102155.39 [78583.44, 147578.55]	137526.54 [104622.00, 194788.05]	125320.50 [95292.45, 174241.27]	144165.24 [100450.40, 191524.46]	<0.001
**Ang-1 (median [IQR])**	809.00 [422.97, 1790.07]	1865.10 [815.37, 3136.87]	996.06 [556.51, 2509.87]	2272.12 [747.62, 4914.04]	<0.001
**Ang-2 (median [IQR])**	12165.87 [6017.92, 22970.81]	5507.53 [2689.95, 10870.70]	7051.79 [2444.12, 14503.13]	4395.15 [2223.28, 8793.63]	<0.001
**Ang-2/-ang-1 (median [IQR])**	13.35 [4.36, 40.03]	3.73 [1.25, 9.93]	6.15 [1.45, 24.34]	1.99 [0.48, 8.49]	<0.001
**E-Selectin (median [IQR])**	29.50 [15.09, 59.73]	24.99 [12.81, 44.56]	26.81 [15.61, 43.92]	26.62 [13.78, 51.75]	0.340
**Fractalkine (median [IQR])**	58.75 [31.10, 148.69]	25.56 [13.95, 56.24]	49.01 [20.64, 125.56]	20.77 [12.75, 41.34]	<0.001
**ICAM (median [IQR])**	258001.90 [182195.54, 430212.25]	189446.56 [121849.54, 301016.48]	248898.25 [144444.37, 384404.06]	201967.60 [117237.69, 319053.97]	<0.001
			**Day 6-8**		
n	34	21	21	79	
**IL-6 (median [IQR])**	49.86 [21.37, 165.60]	27.26 [13.42, 69.53]	44.69 [16.73, 96.38]	24.86 [9.13, 52.42]	0.030
**IL-8 (median [IQR])**	109.06 [45.88, 221.38]	52.01 [29.07, 82.10]	86.24 [39.51, 216.29]	42.11 [21.31, 100.35]	0.008
**IL-10 (median [IQR])**	9.42 [4.88, 17.33]	3.15 [1.47, 6.68]	5.70 [3.44, 15.13]	3.28 [1.60, 6.41]	<0.001
**MMP8 (median [IQR])**	2255.35 [951.82, 3861.82]	864.79 [480.69, 3605.29]	1948.60 [645.26, 4068.04]	1330.16 [588.61, 2405.47]	0.222
**D-Dimer (median [IQR]) ***	17145.00 [9562.30, 20088.00]	10127.00 [7345.70, 13999.00]	18505.00 [8959.40, 24858.00]	11779.00 [5929.90, 21596.50]	0.098
**Protein C (median [IQR])**	123040.31 [97911.90, 170611.63]	164618.51 [127390.68, 205896.88]	137783.78 [98908.67, 210257.14]	167468.88 [122591.83, 214128.86]	0.016
**Ang-1 (median [IQR])**	886.78 [534.92, 2073.90]	1192.84 [754.68, 2677.12]	1051.27 [568.08, 2851.31]	2144.34 [666.22, 5458.76]	0.343
**Ang-2 (median [IQR])**	10094.40 [5622.90, 17420.07]	4445.76 [2164.96, 9123.84]	3326.26 [2453.65, 8508.91]	3737.63 [2756.56, 6627.10]	0.003
**Ang-2/-ang-1 (median [IQR])**	9.75 [3.29, 31.69]	3.33 [1.33, 6.35]	4.04 [0.29, 14.72]	2.29 [0.69, 8.29]	0.013
**E-Selectin (median [IQR])**	45.74 [17.21, 66.04]	32.45 [16.82, 69.09]	32.10 [20.65, 64.24]	27.17 [13.89, 61.13]	0.621
**Fractalkine (median [IQR])**	42.64 [24.16, 99.77]	24.99 [18.63, 48.01]	20.75 [13.37, 94.57]	19.82 [12.75, 39.40]	0.004
**ICAM (median [IQR])**	309338.27 [242274.10, 456972.28]	261894.49 [113760.93, 418716.02]	258187.17 [206502.44, 414920.22]	185529.72 [130378.30, 338427.07]	0.025

Concentrations are in pg/ml, except for *, these are in ng/ml. Abbreviations: ang, angiopoietin; IL, interleukin; MMP, matrix metalloproteinase; ICAM, intercellular adhesion molecule.

Supplementary Table 7. Longitudinal change in the biomarkers in a linear mixed model compared to δ-δ

Biomarker	Comparator	Estimate	95% CI	Interaction p-value unadjusted*
**Biomarkers of inflammation**				
**IL-6**	γ-γ	0.04	0.01-0.07	0.021
	γ-δ	0.02	-0.02-0.06	0.393
	δ-γ	0.02	-0.02-0.06	0.439
**IL-8**	γ-γ	0.04	0.02-0.06	<0.001**
	γ-δ	0.02	-0.01-0.05	0.112
	δ-γ	0.01	-0.02-0.04	0.434
**IL-10**	γ-γ	0.06	0.04-0.08	<0.001**
	γ-δ	0.03	0.00-0.07	0.027
	δ-γ	0.03	-0.00-0.06	0.094
**MMP8**	γ-γ	0.01	-0.02-0.05	0.436
	γ-δ	-0.03	0.08-0.02	0.292
	δ-γ	-0.02	-0.07-0.03	0.434
**Biomarkers of coagulation**				
**D-Dimer**	γ-γ	0.01	-0.00-0.03	0.113
	γ-δ	0.02	-0.00-0.04	0.083
	δ-γ	-0.00	-0.02-0.02	0.949
**Protein C**	γ-γ	0.02	-0.00-0.03	<0.001**
	γ-δ	0.01	0.00-0.02	0.049
	δ-γ	0.01	-0.00-0.02	0.114
**Biomarkers of endothelial dysfunction**				
**Ang-1**	γ-γ	0.01	-0.01-0.02	0.600
	γ-δ	0.01	-0.02-0.03	0.651
	δ-γ	-0.01	-0.03-0.02	0.635
**Ang-2**	γ-γ	0.01	-0.03-0.04	0.635
	γ-δ	-0.05	-0.09-0.00	0.045
	δ-γ	-0.02	-0.06-0.03	0.430
**Ang-2/Ang-1**	γ-γ	0.00	-0.04-0.04	0.929
	γ-δ	-0.05	-0.11-0.00	0.066
	δ-γ	-0.01	-0.06-0.04	0.645
**E-Selectin**	γ-γ	0.01	-0.01-0.02	0.450
	γ-δ	-0.01	-0.03-0.01	0.183
	δ-γ	-0.00	-0.02-0.02	0.773
**Fractalkine**	γ-γ	-0.01	-0.03-0.00	0.043
	γ-δ	0.01	-0.01-0.03	0.164
	δ-γ	-0.02	-0.04-0.00	0.014
**ICAM-1**	γ-γ	-0.01	-0.02--0.00	0.048
	γ-δ	-0.02	-0.03-0.00	0.032
	δ-γ	-0.01	-0.02-0.01	0.199

*Unadjusted for multiple comparison. **Significant after adjustment for multiple comparison. Abbreviations: ang, angiopoietin; IL, interleukin; MMP, matrix metalloproteinase; ICAM, intercellular adhesion molecule.

Supplementary Figure 1. Percentage of the missing variables on ICU admission in the MARS cohort

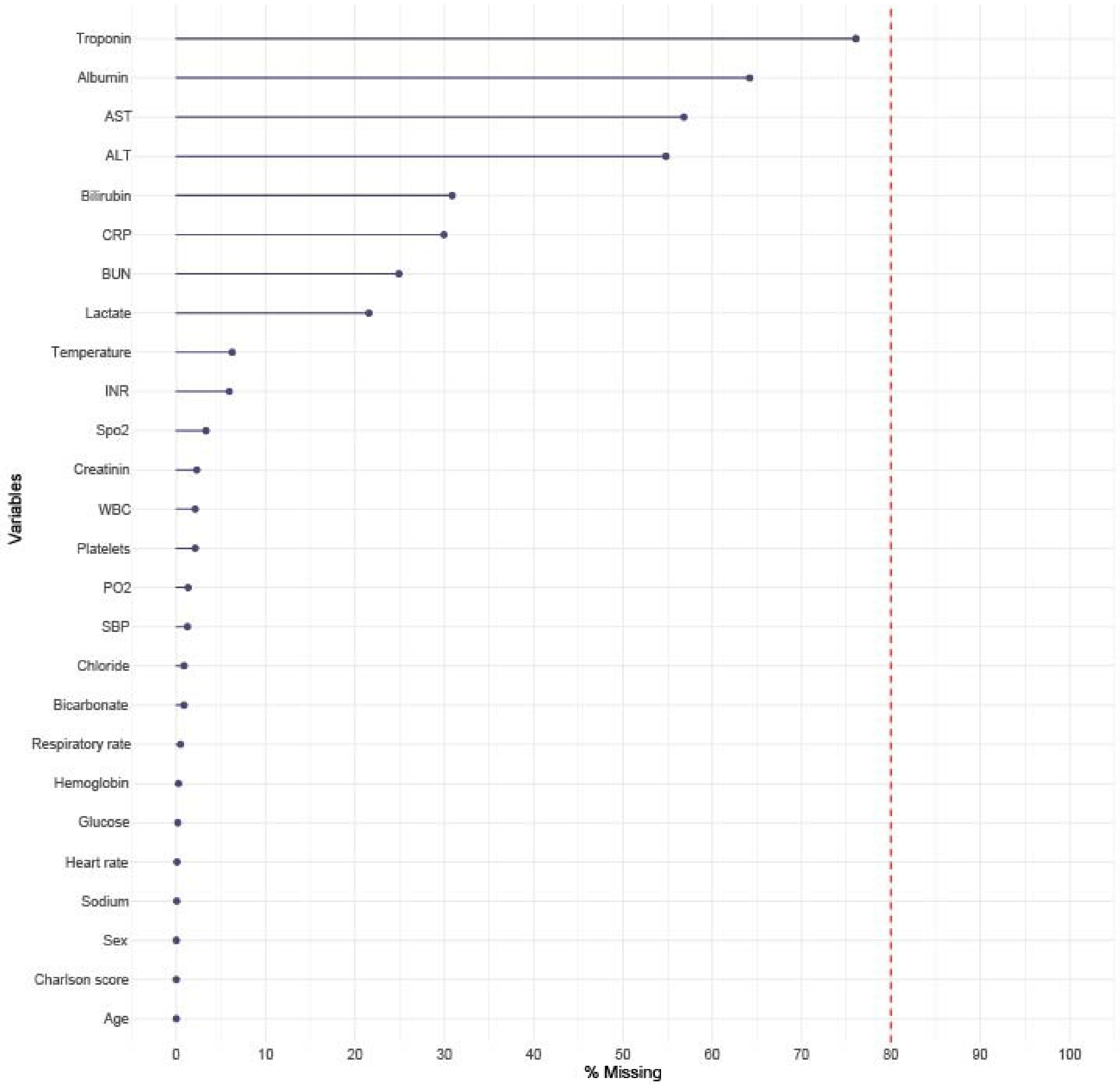

The red dashed line indicates the limit, which was applied to include variables for the subtype adjudication, only variables with <80% missing on ICU admission were used for subtype adjudication. Abbreviations: ALT, alanine transaminase; AST, aspartate aminotransferase; BUN, blood urea nitrogen; CRP, C-reactive protein; INR, international normalized ratio; Po2, Partial pressure of oxygen; SBP, systolic blood pressure; Spo2, oxygen saturation; WBC, white blood cell count.

Supplementary Figure 2. Percentage of the missing variables on day 2, 4 and 7 in the MARS cohort

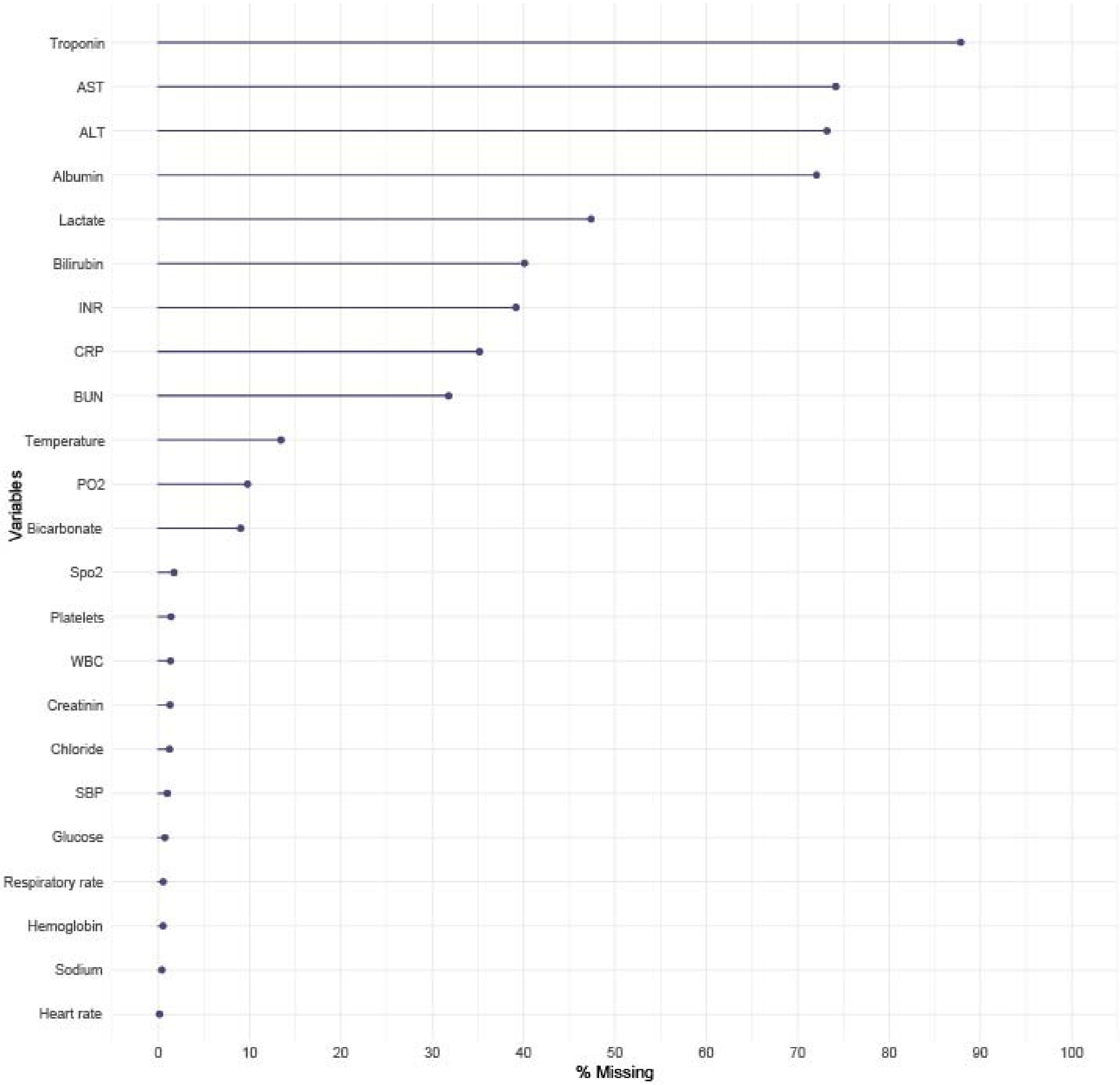

Abbreviations: ALT, alanine transaminase; AST, aspartate aminotransferase; BUN, blood urea nitrogen; CRP, C-reactive protein; INR, international normalized ratio; Po2, Partial pressure of oxygen; SBP, systolic blood pressure; Spo2, oxygen saturation; WBC, white blood cell count.

Supplementary Figure 3. Percentage of the missing variables on ICU admission in the MIMIC-IV cohort

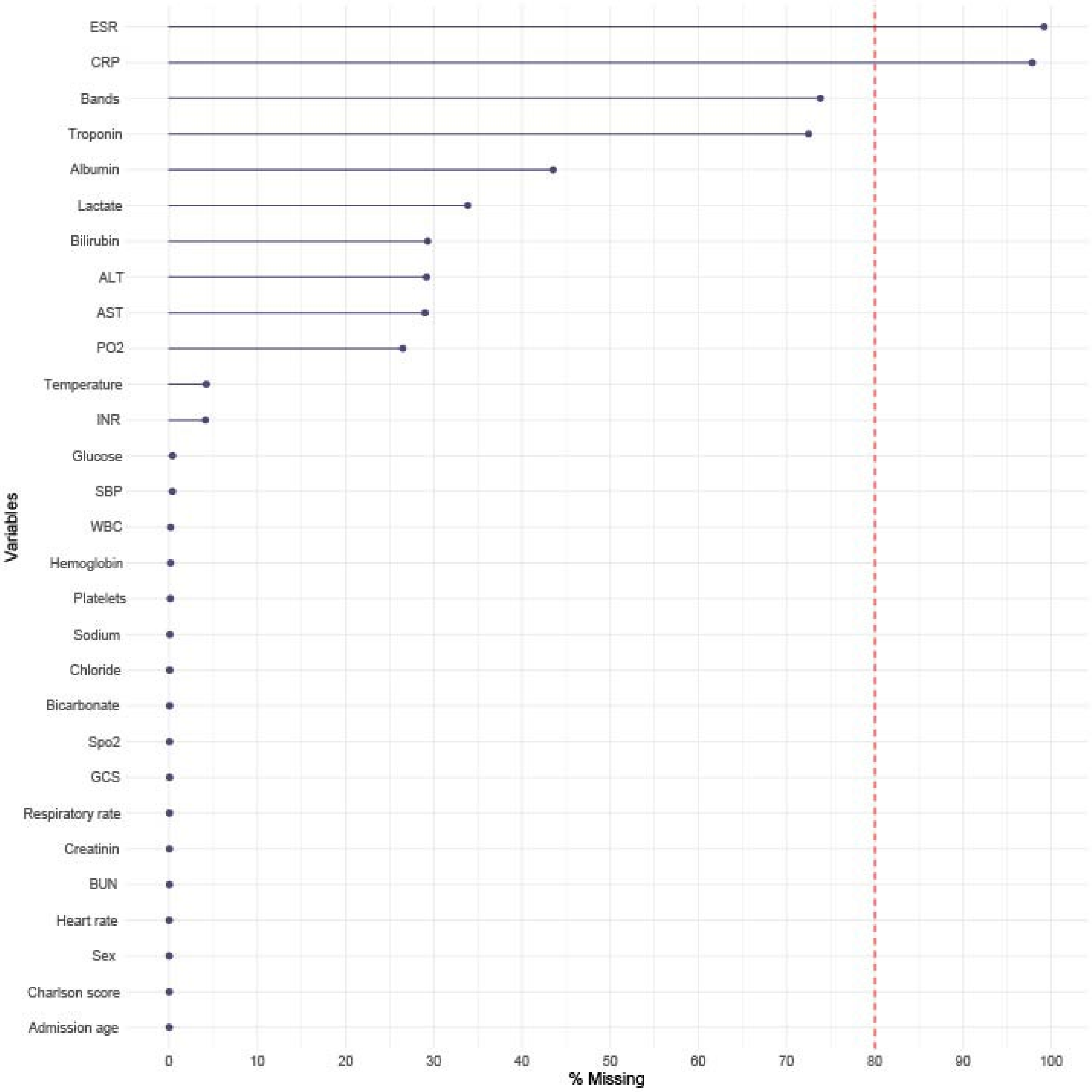

The red dashed line indicates the limit, which was applied to include variables for the subtype adjudication, only variables with <80% missing on ICU admission were used for subtype adjudication; resulting in the exclusion of CRP and ESR. Abbreviations: ALT, alanine transaminase; AST, aspartate aminotransferase; BUN, blood urea nitrogen; CRP, C-reactive protein; ERS, erythrocyte sedimentation rate; GCS, Glasgow Coma Scale; INR, international normalized ratio; Po2, Partial pressure of oxygen; SBP, systolic blood pressure; Spo2, oxygen saturation; WBC, white blood cell count.

Supplementary Figure 4. Percentage of the missing variables on day 2, 4 and 7 in the MIMIC-IV cohort

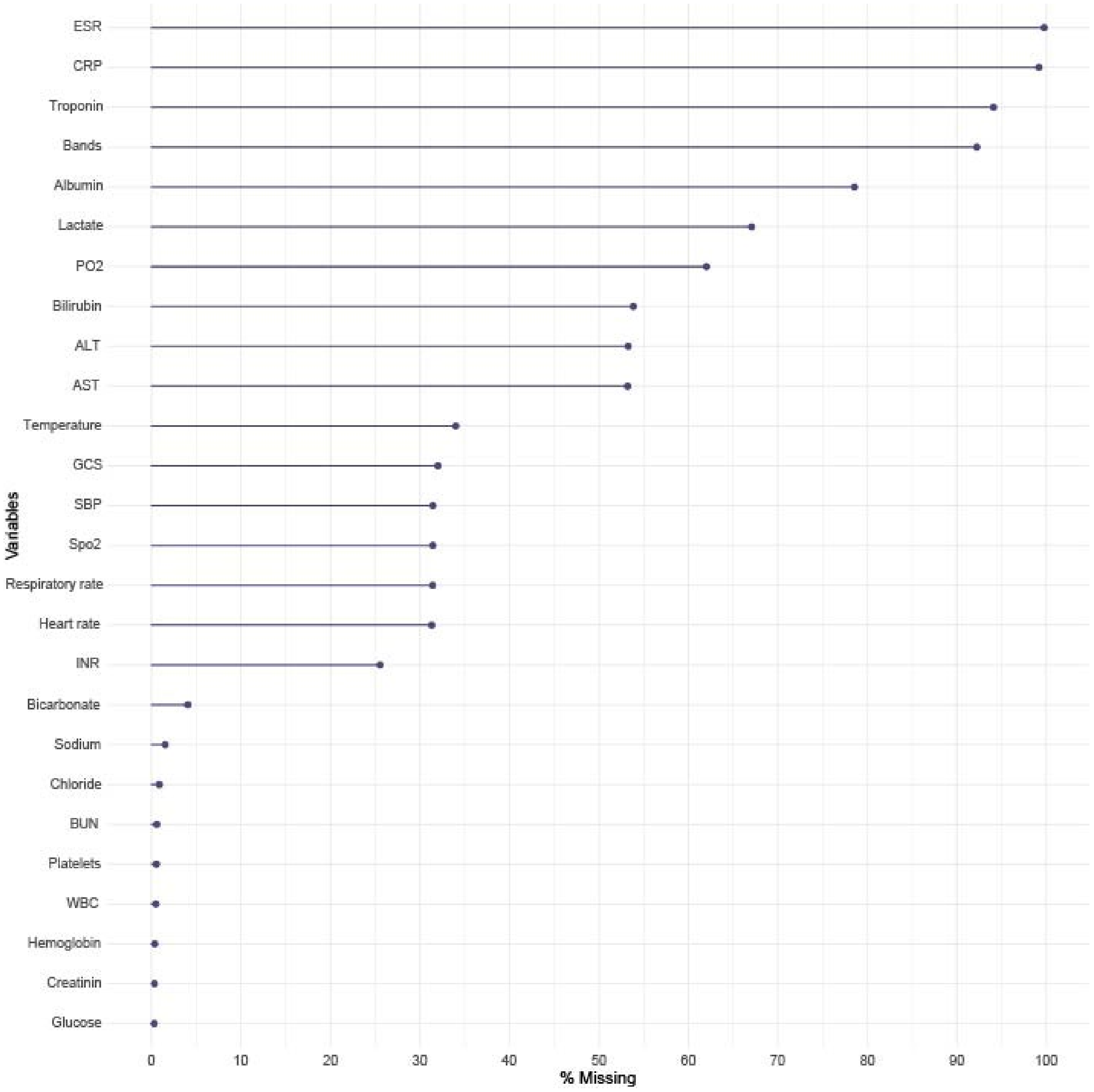

Abbreviations: ALT, alanine transaminase; AST, aspartate aminotransferase; BUN, blood urea nitrogen; CRP, C-reactive protein; ERS, erythrocyte sedimentation rate; GCS, Glasgow Coma Scale; INR, international normalized ratio; Po2, Partial pressure of oxygen; SBP, systolic blood pressure; Spo2, oxygen saturation; WBC, white blood cell count.

Supplementary Figure 5. 30-day survival curves according to the subtypes on ICU admission and day 2 in patients with subtype α or β on admission

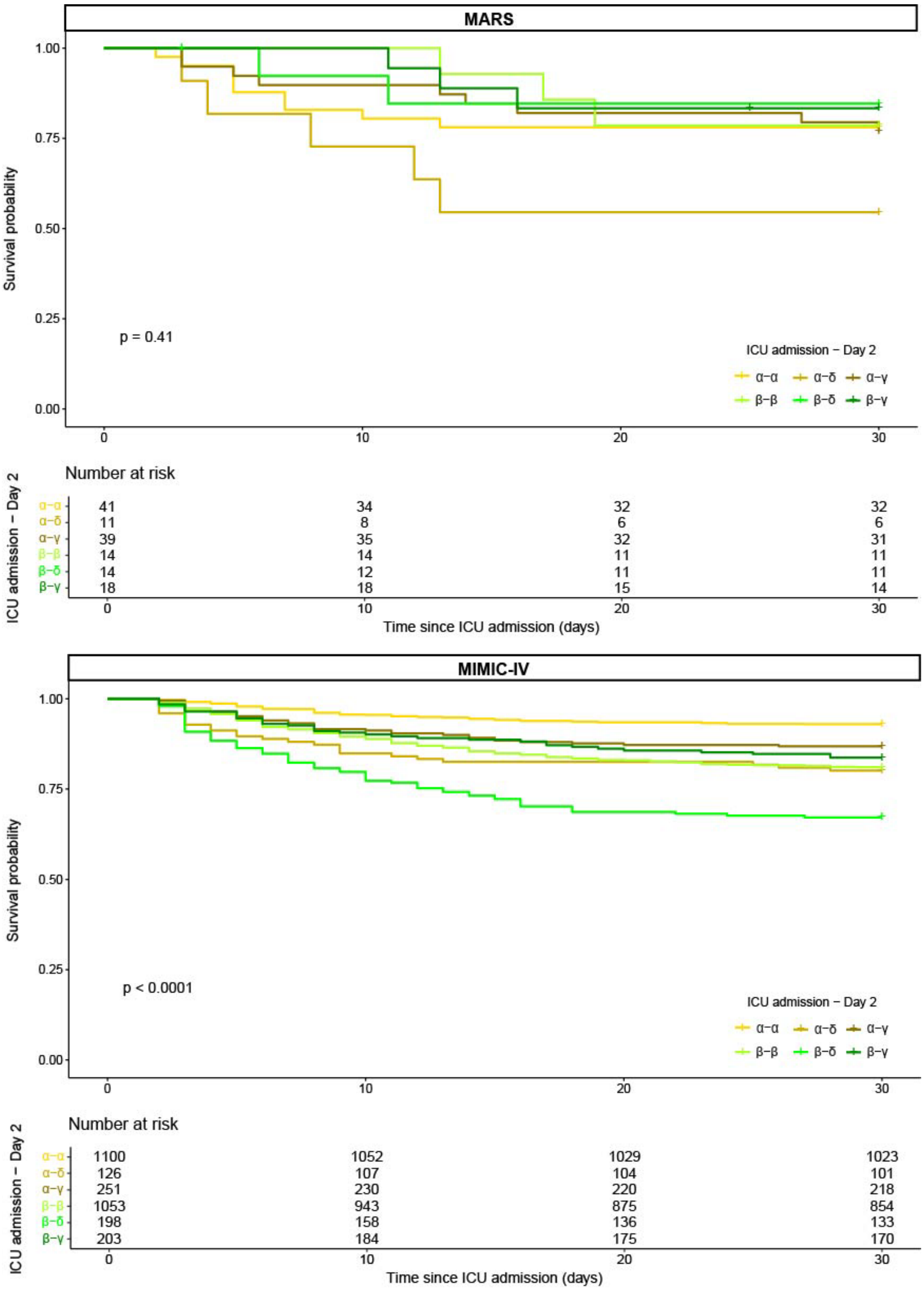

The 30-day survival curves are displayed according to the subtypes on ICU admission and day 2 in the MARS and MIMIC-IV cohort in patients transitioning from α-α to α-γ and α-δ and in β-β to β-γ and β-δ. An unadjusted logistic regression model between the survival curves of the patients with consistent subtype on ICU admission versus those exhibiting a changed subtype by day 2 is shown. Abbreviations: ICU, intensive care unit.

Supplementary Figure 6. 30-day survival curves according to the subtypes on ICU admission and day 4

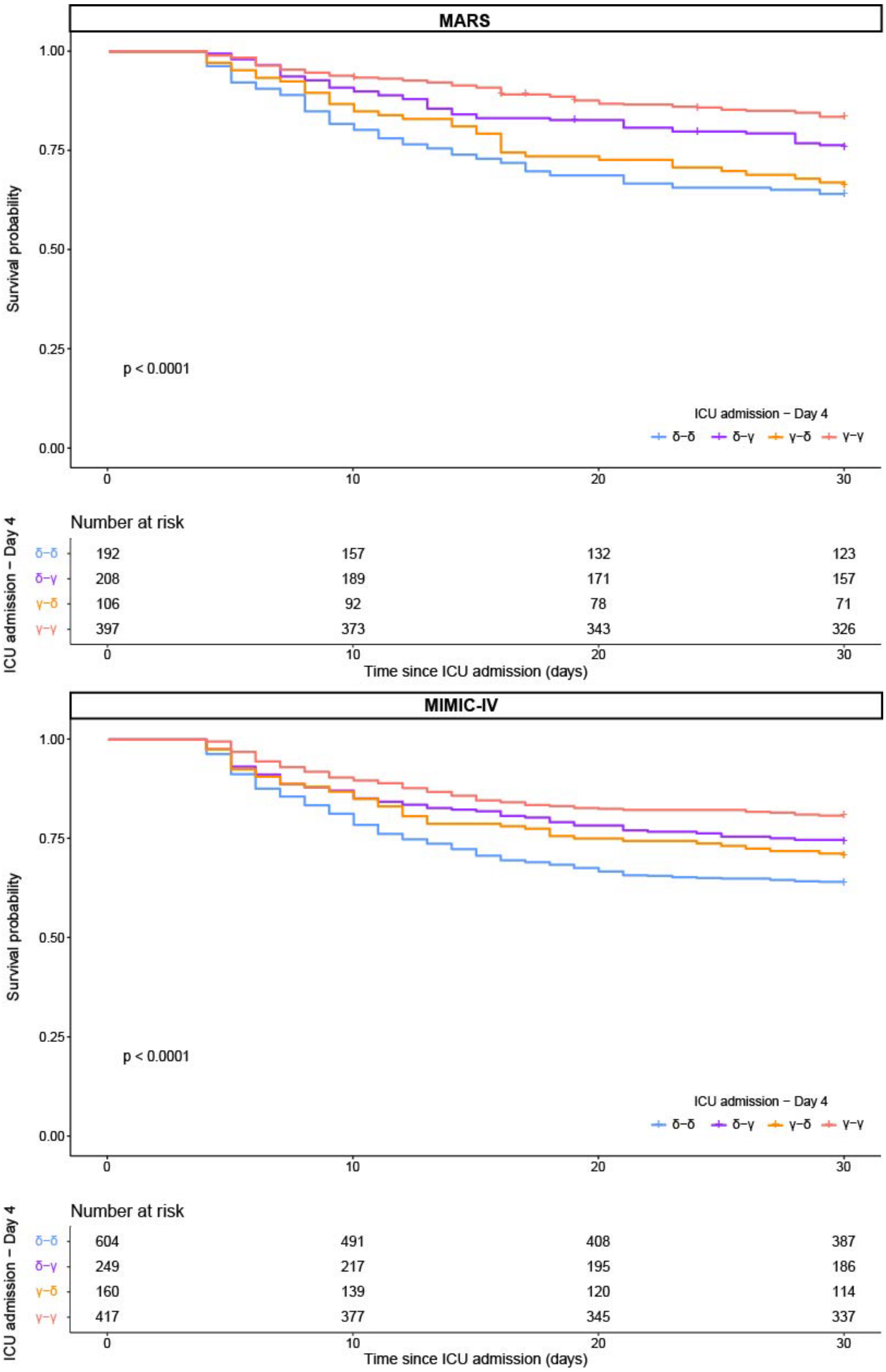

The 30-day survival curves are displayed according to the subtypes on ICU admission and day 4 in the MARS and MIMIC-IV cohort. Only patients with the δ and γ subtype were included in this figure, since the α and γ subtype were only present in a small proportion of the MARS (on baseline α 6.2% and β 2.9%). An unadjusted logistic regression model between the survival curves of the patients with consistent subtype on ICU admission versus those exhibiting a changed subtype by day 4 is shown. Abbreviations: ICU, intensive care unit.

Supplementary Figure 7. 30-day survival curves according to the subtypes on ICU admission and day 7

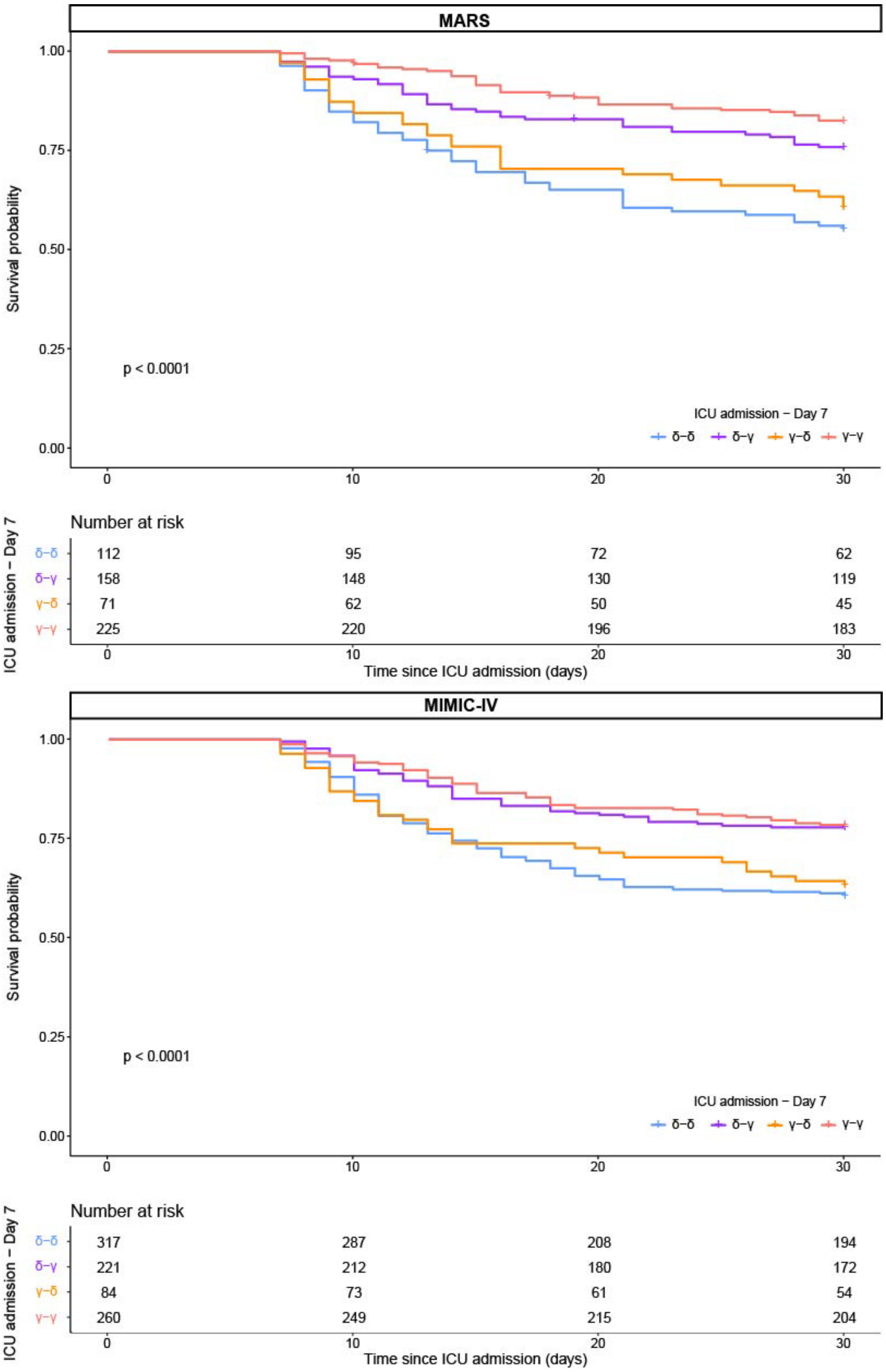

The 30-day survival curves are displayed according to the subtypes on ICU admission and day 7 in the MARS and MIMIC-IV cohort. Only patients with the δ and γ subtype were included in this figure, since the α and γ subtype were only present in a small proportion of the MARS (on baseline α 6.2% and β 2.9%). An unadjusted logistic regression model between the survival curves of the patients with consistent subtype on ICU admission versus those exhibiting a changed subtype by day 4 is shown. Abbreviations: ICU, intensive care unit.

Supplementary Figure 8. Host response biomarkers

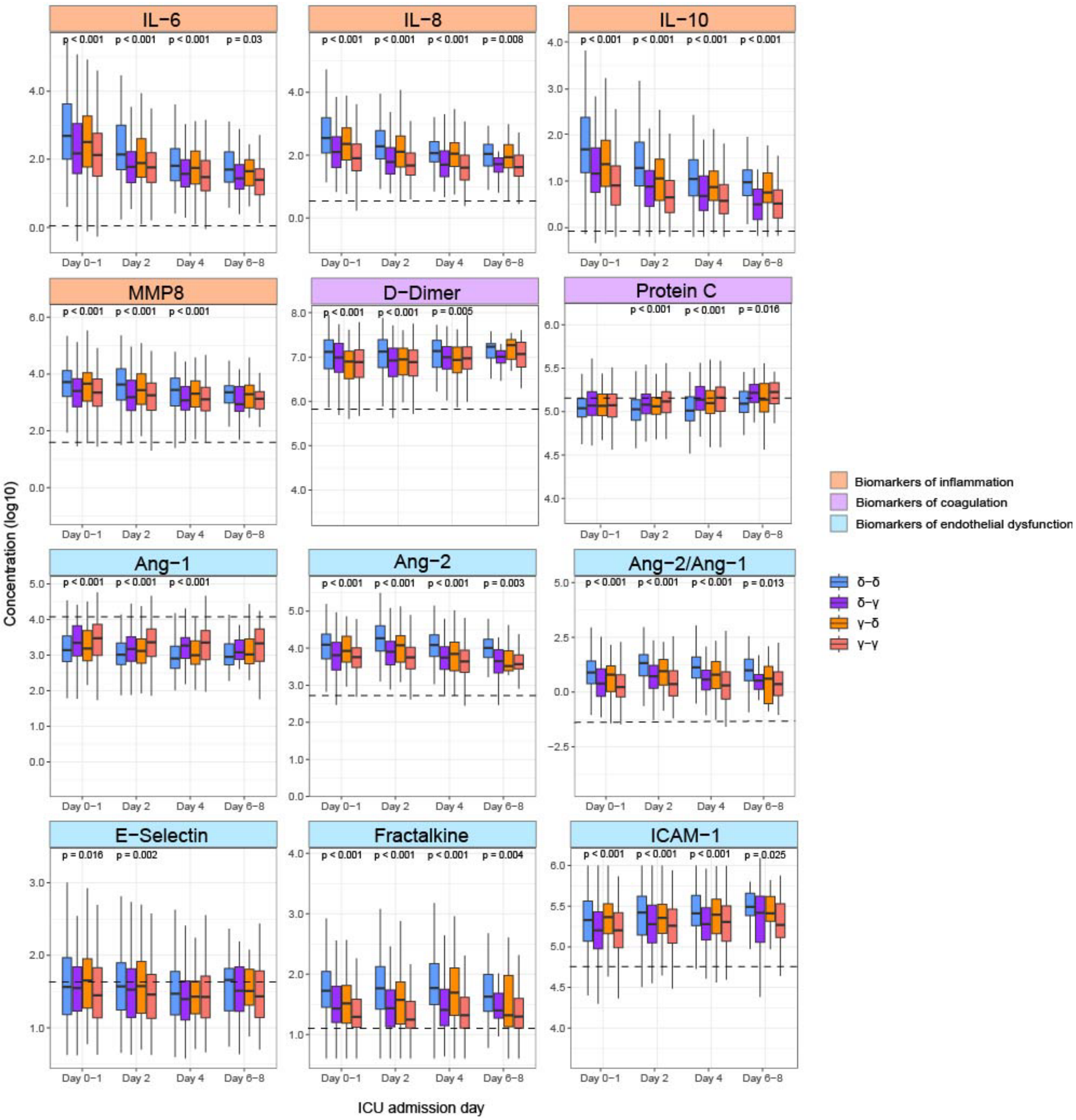

The host response biomarkers during ICU admission are displayed according to the subtypes on ICU admission and day 2 in the MARS cohort. Boxplots displaying biomarker concentrations (pg/ml), after log10 transformation. Horizontal line is the median concentration of healthy volunteers (n = 25). Biomarkers are grouped by domain (Inflammation, Coagulation, and Endothelial dysfunction). Abbreviations: ang, angiopoietin; IL, interleukin; MMP, matrix metalloproteinase; ICAM, intercellular adhesion molecule.

References

1. Zimmerman JE, Kramer AA, McNair DS, Malila FM. Acute Physiology and Chronic Health Evaluation (APACHE) IV: hospital mortality assessment for today's critically ill patients. *Crit Care Med* 2006; **34**(5): 1297-310.
2. Singer M, Deutschman CS, Seymour CW, et al. The Third International Consensus Definitions for Sepsis and Septic Shock (Sepsis-3). *Jama* 2016; **315**(8): 801-10.
3. Garner JS, Jarvis WR, Emori TG, Horan TC, Hughes JM. CDC definitions for nosocomial infections, 1988. *Am J Infect Control* 1988; **16**(3): 128-40.
4. Calandra T, Cohen J. The international sepsis forum consensus conference on definitions of infection in the intensive care unit. *Crit Care Med* 2005; **33**(7): 1538-48.
5. Klein Klouwenberg PM, Ong DS, Bos LD, et al. Interobserver agreement of Centers for Disease Control and Prevention criteria for classifying infections in critically ill patients. *Crit Care Med* 2013; **41**(10): 2373-8.
6. Bellomo R, Ronco C, Kellum JA, Mehta RL, Palevsky P, Acute Dialysis Quality Initiative w. Acute renal failure - definition, outcome measures, animal models, fluid therapy and information technology needs: the Second International Consensus Conference of the Acute Dialysis Quality Initiative (ADQI) Group. *Crit Care* 2004; **8**(4): R204-12.
7. Bernard GR, Artigas A, Brigham KL, et al. The American-European Consensus Conference on ARDS. Definitions, mechanisms, relevant outcomes, and clinical trial coordination. *American journal of respiratory and critical care medicine* 1994; **149**(3 Pt 1): 818-24.
8. Charlson ME, Pompei, P., Ales, K. L., & MacKenzie, C. R. . A new method of classifying prognostic comorbidity in longitudinal studies: development and validation. *Journal of chronic diseases* 1987; **40(5)**: 373–83.
9. Hedges LV. Distribution theory for Glass's estimator of effect size and related estimators. *Journal of Educational Statistics* 1981; **6.2**: 107-28.
10. Brydges CR. Effect Size Guidelines, Sample Size Calculations, and Statistical Power in Gerontology. *Innovation in Aging* 2019; **3**(4).
